# Cell Type-Specific Epigenomic Analysis Reveals a Uniquely Closed Chromatin Architecture in Mouse Rod Photoreceptors

**DOI:** 10.1038/srep43184

**Published:** 2017-03-03

**Authors:** Andrew E. O. Hughes, Jennifer M. Enright, Connie A. Myers, Susan Q. Shen, Joseph C. Corbo

**Affiliations:** 1 Department of Pathology and Immunology, Washington University School of Medicine, St. Louis, Missouri 63110, USA

## Abstract

Rod photoreceptors are specialized neurons that mediate vision in dim light and are the predominant photoreceptor type in nocturnal mammals. The rods of nocturnal mammals are unique among vertebrate cell types in having an ‘inverted’ nuclear architecture, with a dense mass of heterochromatin in the center of the nucleus rather than dispersed clumps at the periphery. To test if this unique nuclear architecture is correlated with a unique epigenomic landscape, we performed ATAC-seq on mouse rods and their most closely related cell type, cone photoreceptors. We find that thousands of loci are selectively closed in rods relative to cones as well as >60 additional cell types. Furthermore, we find that the open chromatin profile of photoreceptors lacking the rod master regulator *Nrl* is nearly indistinguishable from that of native cones, indicating that *Nrl* is required for selective chromatin closure in rods. Finally, we identified distinct enrichments of transcription factor binding sites in rods and cones, revealing key differences in the *cis*-regulatory grammar of these cell types. Taken together, these data provide insight into the development and maintenance of photoreceptor identity, and highlight rods as an attractive system for studying the relationship between nuclear organization and local changes in gene regulation.

Photoreceptors are light-sensitive neurons that express opsins tuned to specific wavelengths of light. In the mouse retina >95% of photoreceptors are rods, which express rhodopsin (*Rho*) and mediate vision in dim light^1^. In contrast, cone photoreceptors express both short- and/or medium-wavelength opsins (*Opn1sw* and *Opn1mw*, respectively) and mediate bright light vision as well as color vision^2^,^3^ (Fig. 1A,B). The rods of nocturnal mammals have evolved a unique nuclear architecture to enhance visual sensitivity in low-light environments^4^. Whereas the nuclei of nearly every vertebrate cell type harbors multiple discrete clusters of heterochromatin largely localized to the nuclear periphery, the rods of nocturnal mammals harbor a dense central core of heterochromatin surrounded by a ring of euchromatin (Fig. 1C).

Emerging evidence indicates that the three-dimensional organization of chromatin within the nucleus is essential for regulating gene expression^5^,^6^. This organization is fundamentally hierarchical: within the nucleus, chromosomes segregate into discrete territories^7^,^8^, and they are partitioned into megabase-scale compartments (similar to euchromatin and heterochromatin)^9^, which are further organized into topologically associating domains (TADs)^10^. Within TADs, architectural proteins (including CTCF, Mediator, and cohesin) direct chromatin looping, mediating local interactions between transcriptional enhancers (i.e., *cis*-regulatory elements, or CREs) and their target genes^11^,^12^. While the unique localization of chromatin in rods has been described extensively at an ultrastructural level^4^, whether or not this higher-order reorganization is correlated with changes in the regulatory landscape of rods (and ultimately gene expression) is not known.

At the molecular level, the inverted nuclear organization of rods depends on the silencing of key nuclear envelope proteins, including lamin B receptor (*Lbr*) and lamin A/C (*Lmna*)^13^. Although it is not clear how these specific loci are regulated in rods, a tremendous amount is known about the transcriptional networks underlying rod and cone identity^14^,^15^. Commitment to a photoreceptor fate is determined early in development by a pair of ‘K50’ homeodomain (HD) transcription factors (TFs)—*Otx2* and *Crx*—whose binding specificity is determined, in part, by a lysine (K) in position 50 of the homeodomain^16^. Beginning at E11.5, *Otx2* provides a necessary signal for photoreceptor specification^17^,^18^. *Otx2* then directly activates *Crx* (first detectable at E12.5), which is also required for photoreceptor maturation^19^,^20^,^21^. Comparative expression profiling of wild-type and *Crx*^−/−^ retinas has shown that *Crx* regulates additional photoreceptor TFs as well as components of the phototransduction machinery^22^,^23^. Furthermore, *Crx*^−/−^ photoreceptors lack outer segments and fail to respond to light^24^. Thus, detailed molecular studies have placed *Crx* at the core of the photoreceptor transcriptional network.

The decision for a photoreceptor progenitor to differentiate into a rod versus a cone photoreceptor is driven by the TF *Nrl*, which is both necessary and sufficient for rod differentiation^25^,^26^. Expression profiling has demonstrated that *Nrl* regulates a large set of rod-specific genes^27^,^28^,^29^, and the rods of *Nrl*^−/−^ mice are transfated into cells with many of the features of native blue cones, including a conventional nuclear architecture with multiple heterochromatin clusters at the periphery^25^,^30^,^31^. In addition to regulating genes that contribute directly to phototransduction, *Nrl* activates additional TFs required for subsets of rod gene expression, including the nuclear receptors (NRs) *Nr2e3*^32^,^33^ and *Esrrb*^34^, as well as the myocyte enhancer factor 2 family member, *Mef2c*^35^.

Expression profiling of wild-type and TF mutant mouse retinas has rapidly advanced the understanding of how photoreceptor TFs are organized into regulatory networks^23^,^28^,^29^,^36^,^37^. In parallel, significant advances have been made in the discovery of retinal CREs across the mouse genome. Specifically, DNase-seq has been used to map regions of open chromatin (containing candidate CREs) in whole retina^38^, and ChIP-seq has been used to elucidate the genome-wide occupancy of individual TFs—CRX^39^, NRL^40^, and MEF2D^41^—also in whole retina. While these studies have proven highly informative, profiling whole retina is limited in that the ascertainment of CREs is biased towards highly abundant cell types, and the specific cell types underlying individual signals is ambiguous.

To begin to elucidate the epigenomic landscape of individual photoreceptor subtypes, we utilized the assay for transposase accessible chromatin using sequencing (ATAC-seq)^42^ to profile purified populations of rods, cones, and *Nrl*^−/−^ photoreceptors. In addition, we performed RNA-seq on flow-sorted rods and *Nrl*^−/−^ photoreceptors, in order to correlate changes in chromatin accessibility with changes in gene expression. Strikingly, we find that mouse rods display a global reduction in open chromatin (relative to native cones as well as >60 additional mouse cell types and tissues profiled by the ENCODE project), and that this reduction depends on *Nrl*. Through comparative analysis of these datasets, we define thousands of photoreceptor class- and subtype-specific candidate CREs, and we find that distinct subsets of these elements are enriched for different sets of transcription factor binding sites (TFBSs). Taken together, these data reveal that *Nrl* mediates a uniquely closed chromatin architecture in rods, and they provide a framework for elucidating the cell type-specific *cis*-regulatory grammar of rods and cones.

## Results

### ATAC-seq of flow-sorted photoreceptors yields cell type-specific maps of open chromatin

**Figure 1:**
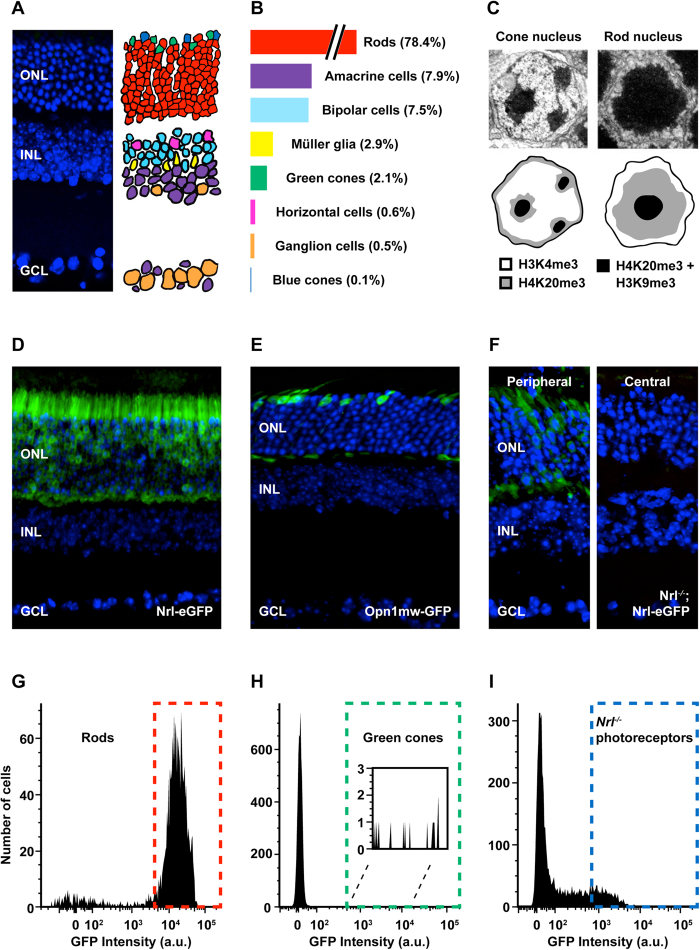
Epigenomic analysis of photoreceptor subtypes. (**A**) The mouse retina is composed of three cellular layers: the outer nuclear layer, the inner nuclear layer, and the ganglion cell layer (ONL, INL, and GCL, respectively). (**B**) There are seven major classes of cells in the retina: rod and cone photoreceptors, bipolar cells, horizontal cells, amacrine cells, ganglion cells, and Müller glia. (**C**) Compared to the nuclei of other cell types (e.g., cones) rod nuclei have an inverted architecture with inactive heterochromatin (H3K9me3, H4K20me3) localized to the center and active euchromatin (H3K4me3) localized to the periphery (image adapted from Corbo *et al*.^39^). (**D**–**F**) Reporter lines used to purify individual photoreceptor types: *Nrl-eGFP* (rod), *Opn1mw-GFP* (green cones), *Nrl*^−/−^*; Nrl-eGFP (Nrl*^−/−^ photoreceptors, or blue cones). In the adult, *Nrl*^−/−^*; Nrl-eGFP* retinas exhibit a peripheral (high) to central (low) gradient of GFP expression (**F**). (**G**–**I**) Representative FACS plots from individual sorts of dissociated retinal cells from each reporter line.

To isolate purified populations of adult mouse photoreceptors, we performed fluorescence-activated cell sorting (FACS) on dissociated retinas harvested from 8-week-old mice harboring transgenic reporter constructs (Fig. 1, Supplementary Table S1). Specifically, we obtained rods from *Nrl-eGFP* mice (Fig. 1D,G)^28^, native ‘green’ cones from *Opn1mw-eGFP* mice (Fig. 1E,H)^43^, and *Nrl*^−/−^ photoreceptors (putatively blue cones) from *Nrl*^−/−^; *Nrl-eGFP* mice (Fig. 1F,I)^28^. We performed ATAC-seq on all three sorted cell types and RNA-seq on sorted rods and *Nrl*^−/−^ photoreceptors, yielding reproducible chromatin accessibility and expression profiles (Pearson correlation coefficients between biological replicates of 0.86–0.99 for ATAC-seq and 0.95–1.00 for RNA-seq) (Supplementary Fig. S1).

**Figure 2:**
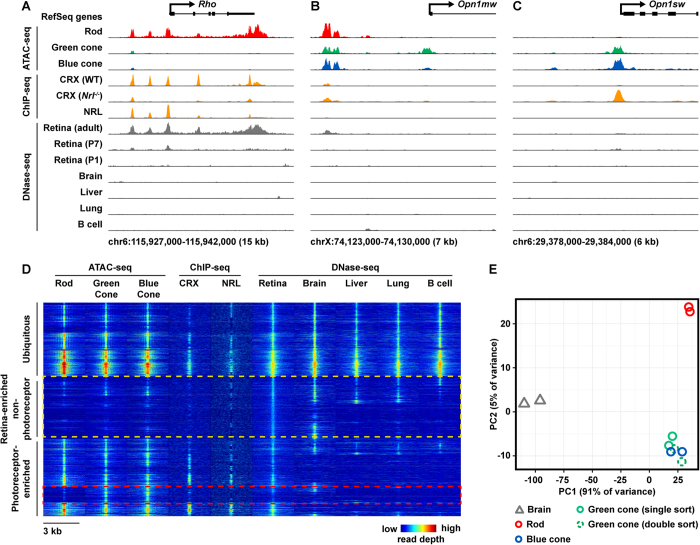
ATAC-seq of flow-sorted cells yields cell type-specific maps of open chromatin. (**A**–**C**) ATAC-seq profile at rod- (**A**) and cone-specific (**B**–**C**) loci. Previously generated data sets also shown: CRX ChIP-seq (photoreceptor-specific TF) in WT (rod-dominant) and *Nrl*^−/−^ (all-cone) retinas, NRL ChIP-seq (rod-specific TF) in WT retina, as well as DNase from whole retina (P1, P7, and adult), brain, liver, lung, and B cells derived from the ENCODE project. (**D**) Genome-wide profile of epigenomic datasets shown in (**C**) across photoreceptor ATAC-seq and adult whole-retina DNase-seq peaks. Rows show the read depth (denoted by pseudo-colored intensity) of the indicated epigenomic dataset in 3 kb windows centered on photoreceptor ATAC-seq and whole-retina DNase-seq peak summits (60,414 peaks randomly down-sampled to 10,000 for plotting). Rows are ordered by hierarchical clustering, revealing that approximately one-third of peaks are ubiquitously accessible (top), one third are non-photoreceptor peaks (middle, yellow box), and one-third are photoreceptor-enriched peaks (bottom). Rod and cone profiles are highly similar, but a subset of cone-enriched peaks appear to be selectively closed in rods (red box). (**E**) Principal component analysis (PCA) of brain DNase-seq and photoreceptor ATAC-seq (two replicates per cell type) shows that the open chromatin profile of rods and cones are highly similar but distinct, whereas cone subtypes are indistinguishable.

ATAC-seq of purified photoreceptors revealed cell type-specific patterns of open chromatin flanking canonical rod- and cone-specific genes: *Rho* (rod-specific), *Opn1mw* (cone-specific), and *Opn1sw* (cone-specific) (Fig. 2A–C). In general, rod ATAC-seq exhibited high concordance with whole-retina DNase-seq (as well as CRX ChIP-seq and NRL ChIP-seq), especially near rod-specific genes (Fig. 2A)^38^,^39^,^40^. This result is expected, given that rods constitute >75% of cells in the mouse retina^1^. In contrast, cones represent only ~2% of cells in the mouse retina. Therefore, we hypothesized that cell type-specific ATAC-seq would be more sensitive than whole-retina DNase-seq for detecting cone-specific regulatory elements. Indeed, ATAC-seq of green cones and *Nrl*^−/−^ photoreceptors revealed many regions of open chromatin that were not previously detected by epigenomic profiling of whole retina, especially near cone-specific genes (Fig. 2B,C). While we observed robust cell type-specific ATAC-seq peaks flanking known rod- and cone-specific genes, we noted that even highly cell type-specific loci frequently harbored peaks open in all three photoreceptor types (Supplementary Fig. S2). This multiplicity of both cell type-specific and shared open chromatin elements in photoreceptors is similar to the ‘locus complexity’ of cell type-specific enhancers recently described by Gonzalez *et al*.^44^.

Although the majority of mouse retinal cells are photoreceptors, we hypothesized that many previously identified whole-retina DNase-seq peaks derive from non-photoreceptor cell types. To compare the chromatin accessibility profiles of flow-sorted photoreceptors and whole retina, we first merged peak calls from cell type-specific ATAC-seq (Supplementary Table S2) and whole-retina DNase-seq to produce a reference set of 60,414 candidate regulatory elements. We then plotted the chromatin accessibility profile of each cell or tissue over these intervals (Fig. 2D). We found that there were many whole-retina DNase-seq peaks that did not correspond to photoreceptor ATAC-seq peaks (Fig. 2D, yellow box). For example, whereas >95% of rod ATAC-seq peaks overlapped whole-retina DNase-seq peaks, <50% of whole-retina DNase-seq peaks overlapped rod ATAC-seq peaks (Supplementary Table S3). To validate that this reduced overlap was due to enhanced specificity (vs. reduced sensitivity), we also examined the overlap of ATAC-seq peaks with photoreceptor-specific ChIP-seq data. We found that rod and cone ATAC-seq peaks overlapped >90% of whole-retina CRX ChIP-seq peaks (i.e., photoreceptor-specific regulatory elements). Furthermore, rod ATAC-seq peaks overlapped >90% of NRL ChIP-seq peaks (i.e., rod-specific regulatory elements). Taken together, these data suggest that photoreceptor ATAC-seq revealed the rod- and cone-specific subsets of open chromatin elements identified by whole-retina DNase-seq, and that a substantial number of candidate CREs identified by whole-retina DNase-seq belong to non-photoreceptor cell types.

We also found that the open chromatin profiles of green cones and *Nrl*^−/−^ photoreceptors were nearly indistinguishable, indicating that the well-described cone-like features of *Nrl*^−/−^ photoreceptors^25^,^30^,^31^ are encoded at the level of chromatin accessibility. This finding was supported by principal component analysis (PCA) as well as hierarchical clustering of the genome-wide open chromatin profiles of photoreceptors and control tissues (Fig. 2E, Supplementary Fig. S3). As *Nrl*^−/−^ photoreceptors preferentially express the short-wavelength opsin (*Opn1sw*), we will henceforth refer to these cells as ‘blue cones’.

### NRL is required for global chromatin closure in rods

**Figure 3:**
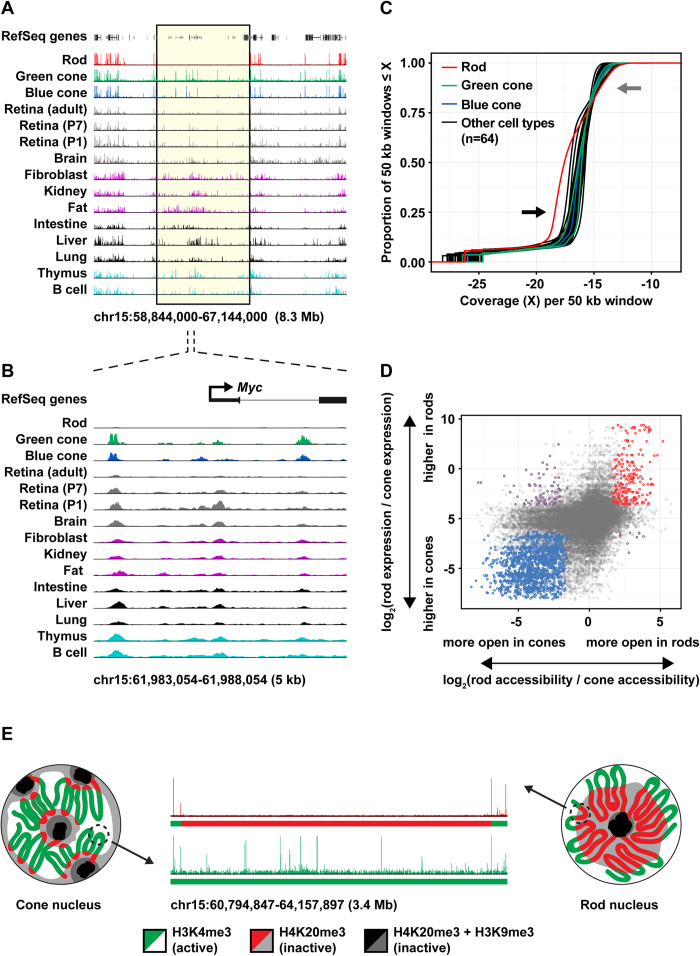
Mouse rods have a uniquely closed epigenomic landscape. (**A**) Representative genomic interval showing an extended run of open chromatin elements selectively closed in rods (yellow box). Tracks show ATAC-seq profiles for rods, green cones, and blue cones and DNase-seq profiles for ten additional tissues (including three developmental time points for whole retina). (**B**) Representative window from (**A**) at higher resolution illustrating rod-specific closure of individual peaks. (**C**) Empirical cumulative distribution functions for genome-wide chromatin accessibility (normalized ATAC-seq or DNase-seq reads in fixed 50 kb windows, see Methods). Rods have a uniquely closed epigenomic landscape relative to blue and green cones as well as 64 additional mouse tissues and cell types (black arrow). Regions that were open in rods tended to have especially high read counts (gray arrow). (**D**) Change in accessibility vs. change in the gene expression in rods and blue cones. For each photoreceptor ATAC-seq peak (n = 55,161), the log of the ratio of normalized ATAC-seq reads (rods/blue cones) is plotted on the x-axis, and the log of the ratio of normalized RNA-seq reads corresponding to the nearest gene (rods/blue cones) is plotted on the y-axis. Peaks that are both significantly differentially accessible (FDR < 0.1) and significantly differentially expressed (FDR < 0.1) are colored red (more open in rods, higher expression in rods), blue (more open in cones, higher expression in cones), or purple (more open in rods, higher expression in cones, or more open in cones, higher expression in rods). Changes in accessibility and expression are directionally correlated, but many peaks near differentially expressed genes are not differentially accessible, and many differentially accessible peaks are not correlated with differential gene expression (especially in cones, left half of plot). (**E**) Schematic proposing how global differences in nuclear organization in rods and cones are correlated with local differences in chromatin accessibility (figure design adapted from Solovei *et al*.^4^).

In our global comparison of rod and cone open chromatin profiles, we noted a significant excess of cone-specific open chromatin relative to rods (Fig. 2D, red box). When we reviewed the genome-wide distribution of these peaks, we found that many occurred in long runs (hundreds of kilobases to tens of megabases), overlapping regions that frequently harbored open chromatin peaks in additional (non-photoreceptor) cell types and tissues (Fig. 3A,B). Therefore, many of the apparently cone-specific regions of open chromatin are more accurately described as regions that are selectively closed in rods. This finding suggested that rods have a more closed chromatin landscape than other cell types and tissues.

To quantify this observation, we examined the distribution of open chromatin signal (ATAC-seq or DNase-seq reads) across the mouse genome in 64 additional cell types and tissues (Fig. 3C, Supplementary Table S4). We divided the genome into fixed 50 kb windows, scored each window for normalized open chromatin signal (normalized ATAC-seq or DNase-seq reads), and then plotted the cumulative distribution (proportion of windows with coverage less than or equal to each observed value) for each cell type or tissue. This analysis revealed that the genome-wide distribution of open chromatin signal in mouse rods was shifted relative to every other cell type and tissue we examined, in a pattern consistent with a more closed chromatin landscape. In *Nrl*^−/−^ photoreceptors, in contrast, these rod-closed regions are open to a similar extent as in other tissues, demonstrating that *Nrl* is necessary for the global chromatin closure phenotype in rods.

We next asked if global differences between the accessible chromatin landscapes of rods and cones were correlated with changes in gene expression. To address this, we mapped individual rod and blue cone ATAC-seq peaks to the nearest transcription start site (TSS) and examined the expression of the corresponding gene (Fig. 3D, Supplementary Tables S5 and S6). In general, changes in chromatin accessibility were directionally correlated with changes in gene expression, i.e., genes near rod-specific peaks tended to have higher expression in rods, while genes near cone-specific peaks tended to have higher expression in cones (Fig. 3D, Supplementary Fig. S4A). We noted, however, that genes near cone-specific peaks had lower expression in both rods and cones relative to genes near rod-specific peaks or shared peaks (Supplementary Fig. S4A), indicating that cone-specific peaks are located in regions that have lower average transcriptional activity in both photoreceptor types. Furthermore, cone-specific open chromatin elements were located significantly farther from annotated genes compared to rod-specific or shared open chromatin elements (median distance 50 kb vs. 18 kb) (Supplementary Fig. S4B). Finally, rod-specific regions of open chromatin were highly enriched for gene ontology (GO) terms related specifically to photoreceptor biology, while cone-specific regions were enriched more generally for terms related to neurodevelopment (Supplementary Fig. S5). Thus, regions of open chromatin that are selectively closed in rods appear to be depleted of genes, especially photoreceptor genes, and have lower transcriptional activity. These findings suggest that rod-specific chromatin closure may not reflect targeted gene silencing, and may instead be secondary to large-scale alterations in nuclear organization (Fig. 3E).

### Lamin A/C expression is selectively downregulated in rods

**Figure 4:**
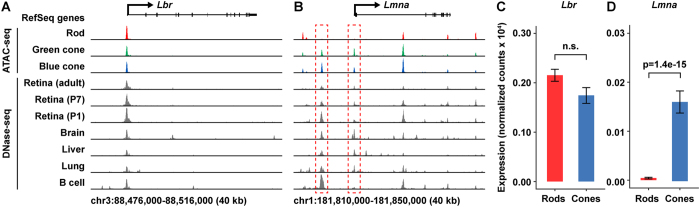
Rods show selective chromatin closure and reduction in gene expression at the *Lmna* but not the *Lbr* locus. (**A**) ATAC-seq profiles from rods, green cones, and blue cones, and DNase-seq profiles from whole retina (P1, P7, and adult), brain, liver, lung, and B cells at the *Lbr* locus. (**B**) Same datasets as in (**A**) shown for the *Lmna* locus. One open chromatin peak overlapping the promoter and one peak ~6.5 kb upstream are selectively closed in rods (red boxes). (**C**) *Lbr* expression in rods and blue cones as measured by RNA-seq. (**D**) *Lmna* expression in rods and blue cones as measured by RNA-seq. For (**C**) and (**D**), bar height corresponds to expression mean (normalized read counts ×10^4^); error bars correspond to ±1 standard deviation. Note that the data in (**C**) and (**D**) are plotted on different scales.

A prior study showed that expression of either lamin B receptor (encoded by *Lbr*) or lamin A/C (encoded by *Lmna*) is required to maintain a conventional nuclear architecture in non-rod cell types^13^. To determine whether expression of these two genes is associated with selective chromatin closure in rods, we examined the open chromatin and transcriptional profiles of these loci in detail (Fig. 4). The *Lbr* locus harbors a single open chromatin peak overlapping the TSS, with comparable ATAC-seq signal in rods, green cones and blue cones (Fig. 4A). It was previously shown by antibody staining that that lamin B receptor is downregulated in both rods and cones as they differentiate^13^. Nevertheless, we detected modest levels of *Lbr* transcript in both rods and blue cones (Fig. 4C), suggesting that either the level of lamin B receptor in adult photoreceptors is regulated post-transcriptionally, or that it is below the limit of detection by antibody staining. While the open chromatin profile surrounding *Lbr* is similar in rods and cones, two peaks at the *Lmna* locus are selectively closed in rods—one overlapping the gene promoter and another ~6.5 kb upstream (Fig. 4B, red boxes). The rod-specific closure of these peaks is correlated with a marked reduction in the level of *Lmna* transcript in rods (Fig. 4D), consistent with the rod-specific reduction in Lamin A/C protein levels reported previously^13^. Taken together, these data indicate that rods selectively downregulate *Lmna* at the transcript level, and this downregulation may be mediated by the selective closure of two upstream open chromatin regions. Furthermore, these findings indicate that NRL mediates chromatin closure at the *Lmna* locus, either directly or indirectly, offering a mechanistic link between the expression of a key rod cell fate determinant and the cell’s inverted nuclear architecture.

### Photoreceptor open chromatin contains combinations of binding sites for photoreceptor TFs

Having characterized photoreceptor open chromatin globally, we next sought to identify local sequence features that mediate the regulatory activity of individual CREs. Combining ATAC-seq peaks from both rods and cones, we detected a total of 55,161 regions of open chromatin in photoreceptors. For each cell type, we partitioned peaks into “promoters” (<1 kb upstream and <100 bp downstream of the nearest TSS) and “enhancers” (>1 kb upstream or >100 bp downstream of the nearest TSS), in light of work demonstrating that cell type-specific regulatory elements are preferentially enriched among TSS-distal elements^45^. Supporting the idea that these regions constitute distinct functional elements, we found that promoter elements in all three photoreceptor types were centered on stronger and broader enrichments of ATAC-seq signal, phylogenetic conservation, and GC content, and that promoter peaks were more strongly correlated with gene expression (Supplementary Fig. S6).

**Figure 5:**
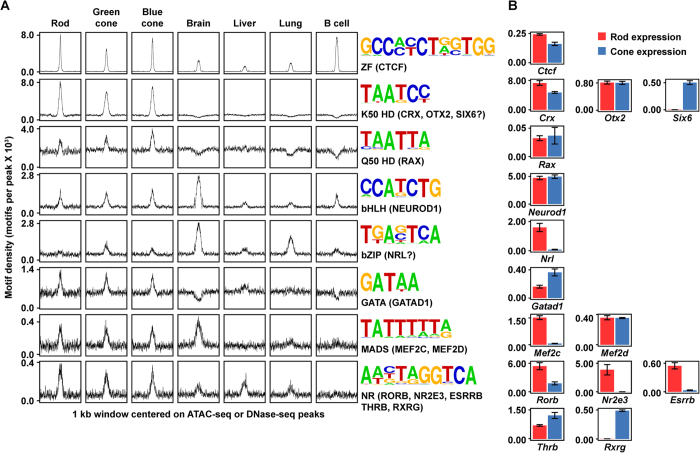
TFBS motif enrichment in photoreceptor enhancers. (**A**) Enrichment of known TFBS motifs in enhancer (TSS-distal) peaks in rods, green cones, blue cones, brain, liver, lung, and B cells. For each panel, distance from peak summit (−500 bp to 500 bp) is plotted on the x-axis and motif density (motifs per peak at each position) is plotted on the y-axis, illustrating central enrichments of the motifs presented. Motifs are labeled by TF class, and candidate photoreceptor TFs that may bind each motif are indicated in parentheses. (**B**) Expression of candidate TFs for each motif in (**A**) in rods and cones as measured by RNA-seq. Bar height corresponds to expression mean (normalized read counts × 10^4^); error bars correspond to ±1 standard deviation.

To identify TFBSs enriched in photoreceptor ATAC-seq peaks, we tested 319 known sequence motifs curated by the HOMER suite of sequence analysis tools for overrepresentation (Fig. 5A, Supplementary Fig. S7, Supplementary Tables S7 and S8)^46^. Whereas promoter peaks were strongly enriched for motifs corresponding to ubiquitous transcriptional regulators (Supplementary Fig. S7), enhancer peaks were enriched for motifs bound by known photoreceptor TFs (Fig. 5A,B). Among enhancer elements in both rods and cones, the most strongly enriched motif corresponded to the zinc-finger (ZF) architectural protein CTCF. CTCF is frequently among the most enriched motifs in open chromatin from most cell types^45^ and is thought to mediate interactions between regulatory elements via chromatin looping^12^,^47^. The second most enriched motif, ‘CTAATCC’, represented the binding of a K50 HD TF, most likely the photoreceptor master regulator CRX^39^. In addition, this motif is also bound by OTX2 (highly expressed during photoreceptor development but at reduced levels in adulthood)^48^ and can be bound by SIX6 as well^49^.

While CTCF and CRX were by far the most enriched motifs in photoreceptor open chromatin, we also observed modest enrichments of additional motifs that again corresponded to binding sites recognized by well-characterized photoreceptor TFs (Fig. 5A,B). These included Q50 HD (TAATTA), basic helix-loop-helix (bHLH) (CATATG), MADS (CC[A/T]_8_GG), NR (AGGTCA), GATA (GATA), and bZIP (TGANTCA) families. The enrichment of non-CRX binding sites is of considerable interest, as it suggests that specific TFs may cooperate with CRX to shape the regulatory activity of photoreceptor-specific CREs. RAX, for example, is the most highly expressed Q50 HD TF (which has glutamine [Q] in position 50 of the homeodomain) in both rods and cones. It has recently been shown that RAX plays an important role in photoreceptor development and is essential for the survival of mature cones^50^. bHLH motifs are likely bound by NEUROD1, which is expressed in developing and mature photoreceptors (both rods and cones)^51^. The MADS family members MEF2D (rods and cones) and MEF2C (rod-specific) are additional essential photoreceptor TFs, and MEF2D in particular has been shown to be recruited by CRX to photoreceptor-specific binding sites^41^. The specific role of individual NR motifs is more complex, as rods and cones express distinct sets of NR TFs—RORB, ESRRB, and NR2E3 are significantly upregulated in rods, while RXRG and THRB are expressed in cones (Fig. 5B). Finally, we observed relative enrichment in cones of a bZIP-type motif (TGANTCA), which may be bound by NRL, a bZIP TF in the MAF subfamily (though MAF family members typically prefer an extended consensus sequence, TGCTGANTCAGCA).

Motifs overrepresented in photoreceptor ATAC-seq peaks were enriched for co-occurrence (overrepresentation of pairs of motifs within individual ATAC-seq peaks) (Supplementary Fig. S8), suggesting they may have cooperative roles in mediating the activity of individual regulatory elements. In particular, we identified a candidate regulatory hub consisting of K50 HD (CRX), NR (RORB), MADS (MEF2D), MAF (NRL), and bZIP (NRL?) motifs that were co-enriched in photoreceptor open chromatin (Supplementary Fig. S8). Notably, while we did not observe a robust enrichment of MAF motifs by themselves, we did observe an enrichment of motif pairs involving MAF motifs. This suggests that NRL may be recruited to rod-specific enhancers cooperatively with additional cell type-specific TFs, analogous to the mechanism of photoreceptor-specific MEF2D occupancy described recently^41^. While overrepresented enhancer TFBS motifs were typically enriched for co-occurrence, peaks harboring CTCF motifs were depleted of other photoreceptor-specific motifs (Supplementary Fig. S8).

Consistent with previous work, we observed modest preferences in spacing and orientation between pairs of enriched motifs^39^ (Supplementary Fig. S9). Nevertheless, specific configurations account for a minority of enriched motif pairs, supporting the idea that photoreceptor regulatory activity is encoded by a flexible sequence grammar, as suggested by previous studies^39^,^52^. Finally, we noted that while the majority of rod and cone ATAC-seq peaks were shared across cell types, many enriched motifs are likely bound by TFs that are differentially expressed in rods and cones *in vivo* (Fig. 5B). This raises the possibility that CREs open in both rods and cones encode cell type-specific regulatory activity via the recruitment of distinct arrays of TFs and/or by competition among differentially expressed TFs (e.g., NRs) for common binding sites. For example, although NRL is a rod-specific TF, >80% (1603/1935) of NRL ChIP-seq peaks overlap elements that are accessible in both rods and cones (Fig. 2D). Thus, differential expression of *trans* factors may be an important mechanism by which photoreceptor regulatory elements encode cell subtype-specific functions within shared *cis*-regulatory elements.

**Figure 6:**
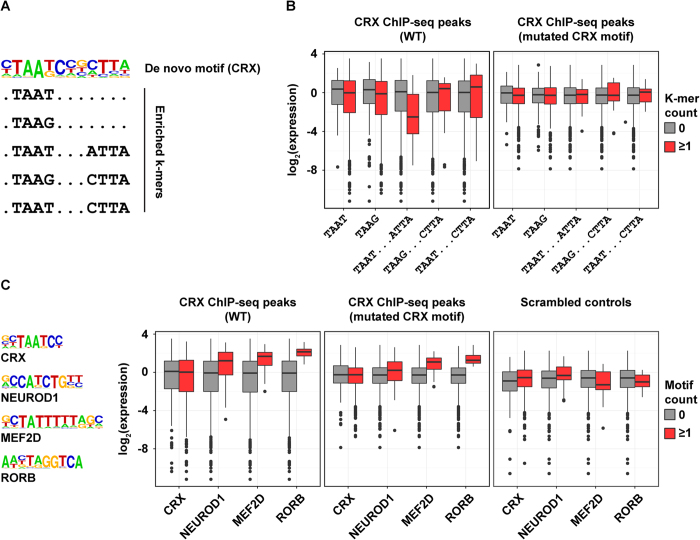
Functional effects of enriched motifs on photoreceptor enhancer activity. (**A**) Dimeric K50 HD motif identified in photoreceptor enhancers (TSS-distal ATAC-seq peaks). The motif logo shows nucleotide preferences scaled to observed frequencies; highly enriched k-mers that compose this motif are listed beneath. (**B**) Expression of CRE-seq constructs from White *et al*. harboring distinct TAAT and TAAG monomeric and dimeric motifs (gray: indicated k-mer not in tested sequence, red: ≥1 instance of indicated k-mer in tested sequence)^52^. The left panel shows the expression of native constructs (84 bp sequences centered on CRX ChIP-seq peaks), and the right plot shows the expression of constructs with CRX motifs eliminated by point mutation (CTAATCC to CTACTCC). (**C**) the expression of constructs assayed by White *et al*. stratified by the presence or absence of four motifs found to be highly enriched in photoreceptor open chromatin: CRX, NEUROD1, MEF2D, and RORB. Box plots show the distribution of expression for constructs with or without the indicated motif in endogenous elements (left panel), constructs with mutated CRX sites (center panel), and scrambled controls (right panel).

In addition to identifying enrichments of known TFBS motifs, we performed *de novo* motif discovery (Supplementary Tables S9 and S10). In general, *de novo* motifs were highly concordant with known motifs that were found to be enriched. A key exception was the *de novo* motif corresponding to a K50 HD family member (likely bound by CRX) (Fig. 6A). While previous *in vitro* studies have shown that CRX exhibits a strong preference for the consensus CTAATCCC^16^, photoreceptor ATAC-seq peaks showed a preference for CRX motifs in a paired configuration on opposite strands separated by exactly three nucleotides (TAAT[N]_3_ATTA), a well-known dimer configuration of HD TFs^53^,^54^. Furthermore, TAAG is highly enriched in photoreceptor open chromatin, especially in a paired configuration (TAAG[N]_3_CTTA or TAAT[N]_3_CTTA). This finding indicates that, in certain contexts, the fourth position of the TAAT homeodomain core tolerates a ‘G’ better than would be expected from quantitative gel shift assays^16^. This conclusion was also reached by a previous study that analyzed hundreds of variants within a single photoreceptor promoter^55^. When we examined individual k-mers underlying the paired K50 HD *de novo* motif, they were highly enriched for the canonical CRX monomer motif (CTAATCC), the 3′ G variant (CTAAGCC), as well as homotypic (TAAT[N]_3_ATTA or TAAG[N]_3_CTTA) and especially heterotypic (TAAT[N]_3_CTTA) dimer configurations (Fig. 6A).

We next asked if distinct configurations of CRX monomeric and dimeric motifs had functional significance with respect to the *cis*-regulatory activity of individual photoreceptor CREs (Fig. 6B). Previously, a library of 84-bp CRX-bound sequences (including 865 WT sequences and 865 versions with CRX binding sites eliminated by point mutation) were assayed for activity in explanted mouse retina via CRE-seq, a massively parallel reporter assay^52^. We re-analyzed these data with respect to CRX motif configuration and did not find strong differences in expression depending on the presence of monomeric (TAAT or TAAG cores), homotypic 3′ G dimeric (TAAG[N]_3_CTTA), or heterotypic dimeric (TAAT[N]_3_CTTA) k-mers. However, we did observe a significant repressive effect for the homotypic TAAT dimer configuration (TAAT[N]_3_ATTA) (Fig. 6B). Furthermore, examining the corresponding control sequences with mutated CRX binding sites, we found that this repressive effect depended on the presence of CRX binding sites (Fig. 6B). Accordingly, these data demonstrate that distinct arrangements of CRX motifs prevalent in endogenous CREs can yield significant differences in *cis*-regulatory activity.

Having identified a repressive effect of TAAT[N]_3_ATTA CRX motifs, we asked if additional motifs enriched in photoreceptor open chromatin contributed to photoreceptor CRE activity. Consistent with the original analysis by White *et al*., we did not observe significant differences in reporter activity among endogenous CRX-bound sequences due to the presence or absence of CRX sites, suggesting the importance of additional sequence features and context (Fig. 6C)^52^. In contrast, we found that constructs harboring bHLH (NEUROD1), MADS (MEF2C and MEF2D), or NR (RORB, NR2E3, and ESRRB) motifs had significantly higher expression compared to those without (Fig. 6C). This activation was retained (though reduced) in control sequences in which CRX sites were eliminated by point mutations, suggesting that CRX may act cooperatively with these TFs to drive enhancer activity. Thus, high-throughput analysis of photoreceptor CREs suggests that specific TFBSs enriched in photoreceptor open chromatin act as potent transcriptional activators *in vivo*.

### Rod- and cone-specific regions of open chromatin are enriched for distinct TFBSs

**Figure 7:**
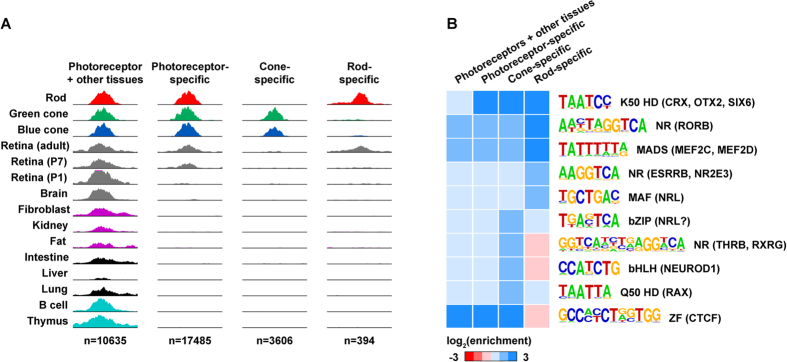
Rods and cones show distinct patterns of TFBS enrichment. (**A**) Examples of peak sets used for motif enrichment analysis: (1) peaks open in photoreceptors and other cell types; (2) photoreceptor-specific peaks (open in rods and cones but not other cell types); (3) cone-specific (open in cones but not rods or other cell types); and (4) rod-specific (open in rods but not cones or other cell types). (**B**) Motif enrichment (ratio of motifs in target vs. background sequences) for selected motifs in the indicated peak sets. Motif logos are labeled by TF family, and cognate photoreceptor TFs are listed in parentheses.

Finally, we asked if rod- and cone-specific ATAC-seq peaks were enriched for distinct sequence features. To define rod- and cone-specific regions, we used DESeq2^56^ to test for differences in accessibility between rods and cones (Methods). This analysis yielded 48,143 shared peaks, 6,324 cone-specific peaks and 693 rod-specific peaks (Supplementary Table S5). To focus on elements likely to contain cell type-specific enhancers, we removed peaks overlapping promoters, peaks shared with non-photoreceptor cell types, and peaks with >70% overlap with repeat sequences. Filtering reduced the totals to 17,485 photoreceptor-specific peaks, 3,606 cone-specific peaks, and 394 rod-specific peaks (Fig. 7A). Thus, we found that the vast majority of photoreceptor-specific peaks were shared between rods and cones, and that there were approximately 10-fold more cone-specific peaks than rod-specific peaks.

As expected, peaks present in non-photoreceptor cell types and tissue showed little enrichment for motifs corresponding to photoreceptor TFs, while shared (rod and cone) photoreceptor-specific peaks were enriched for the factors discussed above (Fig. 7B). Interestingly, rod- and cone-specific peaks were enriched for distinct sets of TF motifs. Specifically, cone-specific open chromatin was enriched for Q50 (RAX), bHLH (NEUROD1), paired NR (THRB and/or RXRG), and a bZIP motif (whose cognate TF is unknown). In contrast, rod-specific open chromatin was specifically enriched for a MAF motif (NRL) and a distinct NR motif (RORB, ESRRB, and/or NR2E3), i.e., motifs bound by rod-specific TFs. Thus, comparative analysis of cell type-specific open chromatin identified specific sequence features that may play an important role in determining rod- vs. cone-specific gene regulation.

## Discussion

In this study, we presented genome-wide maps of open chromatin and gene expression in individual photoreceptor subtypes. These data revealed a striking depletion of accessible chromatin in rods relative to cones and other cell types, which we hypothesize is related to the unique nuclear organization of rods. Furthermore, we leveraged cell type-specific open chromatin maps to identify sequence features that define shared photoreceptor-specific, rod-specific, and cone-specific regulatory elements.

The inverted nuclear architecture of the rods of nocturnal mammals is thought to enhance visual sensitivity in dim light environments^4^. Computational simulations predict that this inverted organization of rod nuclei produces less light scattering than the conventionally organized nuclei of other cell types. Thus, the inverted nuclear architecture confers desirable optical properties to photoreceptor nuclei. In addition, our data suggest that inverted nuclear organization has important implications for the regulatory landscape of mouse rods. Specifically, inverted architecture is correlated with the closure of thousands of regions of open chromatin, which are often clustered within large (10^5^–10^6^ bp) domains and biased towards gene-sparse regions of the genome with lower transcriptional activity in photoreceptors. Thus, while inverted nuclear organization may reflect selection for certain optical properties, precisely how the nucleus is re-packaged may be additionally constrained by the gene expression programs essential for photoreceptor function.

An important outstanding question with respect to inverted nuclear architecture is how it is regulated. As described above, previous work has shown that rod nuclear organization depends on silencing the nuclear envelope proteins *Lbr* and *Lmna*^13^. At the protein level, it was shown that LBR is present in both rods and cones in early postnatal life, and downregulated in both cell types as they mature. Furthermore, as LBR is downregulated, Lamin A/C protein is selectively upregulated in cones (and other retinal cell types) but not rods. In the current study, we find low levels of *Lmna* transcript present in adult cones but not rods, suggesting that rods selectively inhibit *Lmna* transcription. In addition, we find two open chromatin peaks (candidate regulatory elements) at the *Lmna* locus that are selectively closed in rods. We note that these peaks are open in both endogenous cones and *Nrl*^−/−^ photoreceptors, indicating that *Nrl* mediates selective closure of these elements, though additional experiments are needed to determine if this is a direct or indirect effect. Nevertheless, these observations constitute a first step towards localizing changes in the expression of key nuclear envelope genes within rod-specific gene regulatory networks.

We previously showed that acute knockout of *Nrl* results in a partial conversion of rods into cones and can delay photoreceptor degeneration in a mouse model of retinitis pigmentosa^57^. The present study suggests that widespread chromatin closure in adult rods may represent an epigenetic barrier to complete rod-to-cone reprogramming after acute *Nrl* knockout. It will be interesting to determine whether the dynamics of chromatin closure in developing rods temporally correlates with the acquisition of resistance to reprogramming observed in our prior study. Furthermore, if rod-specific chromatin closure is indeed related to the cell’s inverted nuclear architecture, adult human rods, which have a conventional chromatin architecture, may prove more responsive to direct reprogramming strategies than mouse rods.

Recently, Mo *et al*. also evaluated the cell type-specific epigenomic profile of mouse rods^58^. In particular, Mo *et al*. identified differential DNA methylation in rods and cones, which revealed a striking rod-specific enrichment of hypomethylated DNA in closed chromatin. Mo *et al*. suggest that many of these regions represent ‘vestigial enhancers’—regulatory elements active earlier in development that retain hypomethylation marks in adult cell types despite loss of activity^59^. This is consistent with our observations that these regions appear to be less transcriptionally active in both adult rods and cones, and that cone-specific open chromatin is more strongly enriched for GO terms related to neural development than photoreceptor physiology. Finally, while we show that *Nrl* is required for chromatin closure in rods, Mo *et al*. also profiled *Nr2e3*^−/−^ retinas, which demonstrate an intermediate epigenetic phenotype between that of *Nrl*^−/−^ and WT retinas. This finding suggests that rod-specific nuclear organization depends at least in part on TFs downstream of *Nrl.*

In addition to defining the genome-wide chromatin accessibility profile of individual photoreceptor subtypes, our analysis suggests a simple taxonomy of photoreceptor regulatory elements based on TF motif content and their proximity to genes. Consistent with previous work, we found that promoters and enhancers are distinct with respect to size, sequence content and specificity. In particular, promoters tend to be shared across cell types and are enriched for binding sites corresponding to ubiquitous transcriptional regulators, whereas enhancers exhibit greater cell type specificity and are enriched for binding sites corresponding to photoreceptor TFs. Furthermore, enhancers are highly enriched for CTCF binding sites, most of which are shared with non-photoreceptor cell types (likely reflecting ubiquitous TADs), though some are photoreceptor-specific (potentially mediating cell type-specific contact domains). Enhancers containing CTCF motifs were largely devoid of motifs for photoreceptor TFs, whereas enhancers without CTCF motifs are highly enriched for the latter. Taken together, these findings suggest that CREs can be divided into three classes: promoters, CTCF-bound enhancers, and non-CTCF-bound enhancers. We suggest that this tripartite classification is applicable to other cell types.

With respect to non-CTCF-bound enhancers, our motif enrichment analysis identified a set of candidate TFBSs that extend the ‘vocabulary’ of photoreceptor regulatory elements beyond K50 HD (CRX) binding sites, suggesting key roles for Q50 HD, bHLH, NR, MADS, MAF and bZIP TF family members. In parallel, our expression analysis identified TFs that were robustly expressed in rods and/or cones that likely bind these motifs. With respect to K50 HD TFs, we noted that *Six6* is expressed at markedly higher levels in adult cones than in rods. While *Six6* is essential for retinal development in vertebrates^60^,^61^,^62^, a photoreceptor-specific role for *Six6* has not been described in mammals. Nevertheless, *Six6* has been shown to mediate photoreceptor differentiation in medaka^63^, and a closely related homolog, *Six7*, has recently been shown to regulate cone-specific gene expression and survival in zebrafish^64^,^65^. Additional work is needed to determine if *Six6* plays an analogous role in mouse cones.

In addition to identifying binding sites for TFs that likely cooperate with CRX, our analysis suggests that CRX binding sites themselves harbor considerable complexity, including both monomeric and dimeric configurations with distinct nucleotide preferences. That key photoreceptor enhancers contain multiple K50 HD sites capable of binding CRX dimers has been shown previously^22^,^66^. Here, we show that this paired configuration is widespread among photoreceptor regulatory elements. This is particularly intriguing in light of recent work showing that individual nucleotides within paired K50 and Q50 homeodomain motifs (employing the same spacing and orientation preferences found in mouse photoreceptors) dictate photoreceptor subtype-specific gene expression in *Drosophila*^67^. Comprehensive functional analysis will be required to determine if mammalian photoreceptors exploit a similar *cis*-regulatory logic to control photoreceptor subtype-specific expression. Such analyses in primary photoreceptors (both in culture and *in vivo*) are now possible given recent innovations in sequencing-based multiplex reporter assays^52^,^55^,^68^,^69^.

We note that while nearly all motifs enriched in photoreceptor enhancers corresponded to known photoreceptor TFs, we were unable to unambiguously assign the cone-specific enrichment of non-MAF bZIP motifs (TGANTCA) to a candidate regulator. Nevertheless, we suggest that NRL might mediate selective closure of these elements in rods, given that bZIP TFs, including NRL, bind DNA as flexible homotypic or heterotypic dimers that tolerate a range of binding sites. NRL in particular has been shown to bind non-MAF bZIP sites as a heterodimer with either Fos or Jun^70^. This raises the intriguing possibility that NRL has opposing activities (activation vs. repression) at distinct classes of bZIP sites *in vivo*—either autonomously or in combination with non-cell type-specific bZIP TFs.

Overall, the patterns of motif enrichment we describe yield comprehensive, single-nucleotide resolution maps of regulatory sequence features that can inform functional studies of photoreceptor-specific enhancer activity. These data suggest that—even when comparing highly related cell types—cell type-specific regulatory activity is encoded by complex interactions among shared and cell type-specific TFs acting cooperatively and/or competitively to bind target sequences. Thus, a complete understanding of cell type-specific regulatory logic will likely require an integrated analysis of both *cis-* and *trans*-acting factors.

## Materials and Methods

### Mice

Mouse husbandry and all procedures (including euthanasia by CO_2_ inhalation and cervical dislocation) were conducted in accordance with the Guide for the Care and Use of Laboratory Animals of the National Institutes of Health, and were approved by the Washington University in St. Louis Institutional Animal Care and Use Committee.

### Histology

Retinas were harvested from adult (8-week-old) mice, fixed in 4% paraformaldehyde in phosphate buffered saline (PBS) overnight at 4 °C, equilibrated in 30% sucrose in PBS, embedded in Tissue-Tek O.C.T (Sakura), and stored at −80 °C until sectioning. 14 μm cryosections were prepared at −20 °C. Imaging was performed with a Zeiss LSM 700 confocal microscope.

### Retinal dissociation and FACS

Retinas were harvested from adult (8-week-old) male mice unless otherwise noted (Supplementary Table S1) and dissociated with trypsin as described previously^71^. Dissociated cells were resuspended in sorting buffer (1% fetal bovine serum [FBS], 0.1 mM ethylenediaminetetraacetic acid [EDTA] in calcium- and magnesium-free Hank’s buffered salt solution [HBSS]). Cells were sorted using a FACSAria II (BD Biosciences). Single, viable cells were isolated by gating on forward scatter, side scatter, and pulse width. GFP-negative retinas were included in each sort as negative controls. GFP-positive cells were collected in 10% FBS in PBS (for ATAC-seq) or Buffer RLT (RNeasy mini kit, Qiagen) (for RNA-seq). For green cones, we found that double-sorting was necessary to achieve optimal purity. For these sorts, cells were first collected in sort buffer and then re-sorted and collected in 10% FBS in PBS.

### ATAC-seq

ATAC-seq was performed as described previously^42^. Briefly, 10,000–50,000 sorted cells were pelleted by centrifugation (500 g for 5 min at 4 °C), washed twice with PBS, and nuclei were harvested by cold NP-40 lysis. Nuclei were incubated with 2.5 μl of Tn5 transposase (Nextera, Illumina) in a 50 μl reaction volume at 37 °C for 30 minutes. Tagmented DNA was then purified using a MinElute PCR Purification kit (Qiagen). Library fragments were amplified with Phusion High-Fidelity DNA Polymerase (NEB), with the total number of PCR cycles calibrated by parallel qPCR reactions. Libraries were purified using PureLink PCR Purification kits (Invitrogen). Library quality was assessed by gel electrophoresis, and final libraries were quantified using KAPA Library quantification kits (Kapa Biosystems). Libraries were pooled in equimolar ratios and run on an Illumina HiSeq 2500 (paired-end 50 bp reads) (Supplementary Table S1).

### RNA-seq

Library preparation was performed using 5 ng of total RNA. RNA integrity was assessed using an Agilent Bioanalyzer. cDNA was prepared using the SMARTer Ultra Low RNA kit for Illumina Sequencing-HV (Clontech) per manufacturer’s instructions. cDNA was fragmented using a Covaris E210 sonicator using duty cycle 10, intensity 5, cycles/burst 200, time 180 seconds. cDNA was blunt-ended, had an ‘A’ base added to the 3′ ends, and then had Illumina sequencing adapters ligated to the ends. Ligated fragments were amplified for 12 cycles using primers incorporating unique index tags. Replicate libraries from both cell types were pooled in equimolar ratios and sequenced on an Illumina HiSeq 2500 (single-end 50 bp reads) (Supplementary Table S1).

### ATAC-seq data processing

Paired-end ATAC-seq reads were aligned to the GRCm38/mm10 mouse genome assembly using Bowtie2 (v2.2.3) in end-to-end mode with a maximum fragment size of 2000^72^. Alignments were filtered to remove reads with mapping quality <30, discordant read pairs, reads aligning to the mitochondrial genome, and reads aligning to ENCODE blacklisted regions^73^ using SAMtools (v1.3)^74^. PCR duplicates were removed using Picard (v1.121) (http://picard.sourceforge.net). Finally, we removed alignments with an insertion size greater than 100 bp to enrich for nucleosome-free reads (NFR).

For visualization in the UCSC genome browser, bedgraph files were generated from NFR alignments for pooled replicates using HOMER (v4.8), specifying a maximum file size of 500 Mb^46^. Reproducible peaks were called for each cell type using MACS2 (v2.1.0) (using a 100 bp shift and a 200 bp extension, and calling subpeaks)^75^ with an irreproducible discovery rate (IDR) threshold of 0.01^76^. ‘ATAC-seq peaks’ described in subsequent analysis refer to 200 bp elements centered on peak summits. To assess reproducibility between biological replicates, read counts were normalized using the median-of-ratios method^77^, and we then plotted log_2_(normalized read counts +1) for each peak for each replicate (Supplementary Fig. S1) and calculated the Pearson correlation coefficient (PCC) between replicates. This workflow was used for ATAC-seq data generated in the current study, as well as previously generated ATAC-seq data presented in Fig. 3C: pre-B cells (GSE63302)^78^, activated B cells (GSE71698)^79^, and purified neurons (GSE63137)^80^.

### RNA-seq data processing

Single-end RNA-seq reads were aligned to GRCm38/mm10 with STAR (v2.4.2a), using an index prepared for 50 bp reads and the RefSeq gene model^81^. Read counts per gene were calculated using HTSeq^82^. To assess reproducibility between biological replicates, read counts were normalized using the median-of-ratios method^77^, and we then plotted log_2_(normalized read counts +1) for each gene for each replicate (Supplementary Fig. S1) and calculated the Pearson correlation coefficient (PCC) between each pair of replicates.

For visualization in the UCSC genome browser, bedgraph files were generated for pooled replicates using HOMER (v4.8), specifying a maximum file size of 500 Mb^46^.

### ENCODE DNase-seq data processing

FASTQ files from DNase-seq datasets generated by ENCODE were downloaded from the ENCODE data portal (https://www.encodeproject.org/) and processed identically to ATAC-seq data except that single-end reads were used when paired-end reads were not available and alignments were not filtered for NFR reads (insert size <100 bp). Individual datasets and accessions are listed in Supplementary Table S4.

### ChIP-seq data processing

FASTQ files from previously generated ChIP-seq datasets were downloaded from GEO and processed identically to ATAC-seq data except that single-end reads were used when paired-end reads were not available, alignments were not filtered for NFR reads (insert size < 100 bp), and peaks were called on ChIP-seq replicates (vs. input control) with MACS2 (v2.1.0) using default parameters and an FDR of 0.01. These data are presented in Fig. 2A–D and Supplementary Table S3: CRX ChIP-seq (GSE20012)^39^ and NRL ChIP-seq (https://datashare.nei.nih.gov/nnrlMain.jsp)^40^. Two biological replicates were pooled for both WT and *Nrl*^−/−^ CRX ChIP-seq. A single biological replicate of NRL ChIP-seq was run on an Illumina platform (Genome Analyzer). Only this replicate was included in our analysis of NRL ChIP-seq data.

### Quantification of data over photoreceptor ATAC-seq peaks

ATAC-seq, DNase-seq, and ChIP-seq read depths as well as phylogenetic conservation (phastCons 60-way vertebrate conservation)^83^, and GC content were quantified over peak sets using HOMER (v4.8)^46^. For per-feature count tables (used to generate the heatmap in Fig. 2D), photoreceptor ATAC-seq and adult whole-retina DNase-seq peaks were combined using BEDOPS (v2.4.14)^84^, and the indicated datasets were scored over these intervals in 5 bp bins distributed over a 3 kb window centered on peak summits. For average signal histograms (Supplementary Fig. S6), data were scored separately for rod, green cone, and *Nrl*^−/−^ photoreceptor ATAC-seq peaks in 5 bp bins distributed over a 2 kb window centered on the nearest TSS (promoter peaks) or peak summits (enhancers).

### Quantification of sample relatedness

For PCA of brain DNase-seq and photoreceptor ATAC-seq, peaks from each cell type were merged into a master list with BEDOPS (v2.4.14)^84^. For each replicate, reads were counted within elements of the master list using bedtools (v2.24.0)^85^. PCA was then performed on regularized logarithm-transformed values^56^. For pairwise analysis between replicates of photoreceptor ATAC-seq and whole retina, brain, lung, liver and B cell DNase-seq, peaks were again combined using BEDOPS (v2.4.14)^84^. Elements in the union were scored for coverage in each replicate using bedtools (v2.24.0)^85^. We then calculated the pairwise Spearman correlation coefficient (ρ) matrix, clustering rows and columns by 1-ρ using average linkage.

### Comparative analysis of global chromatin closure

To quantify the global distribution of chromatin accessibility, we partitioned the genome into 50 kb fixed windows and calculated read coverage over these windows for each cell type and tissue using bedtools (v2.24.0)^85^. For each sample, we normalized counts to total reads, and plotted the empirical cumulative distribution (proportion of 50 kb windows with less than or equal to each observed coverage value).

### Identification of differentially accessible peaks

ATAC-seq peaks from each photoreceptor type were merged into a master list with BEDOPS (v2.4.14)^84^. For each ATAC-seq replicate, reads were counted within elements of the master list using bedtools (v2.24.0)^85^, and differentially accessible peaks were identified with DEseq2 testing for a log_2_(fold change) greater than 1 at an FDR of 0.1^56^. Counts from rods, double-sorted green cones, and *Nrl*^−/−^ photoreceptors were used for differential accessibility analysis, with cone subtypes collapsed to a single level. The results of this analysis were used to partition photoreceptor ATAC-seq peaks into three subsets: “rod-specific” (significantly more open in rods), “cone-specific” (significantly more open in cones), and “shared” (differential accessibility not statistically significant).

### Identification of differentially expressed genes

We used DEseq2 to test for differential expression between rods and cones using per gene read counts for rod and *Nrl*^−/−^ photoreceptor RNA-seq data^56^. Statistical testing was performed using a log_2_(fold change) threshold of 1 and an FDR of 0.05.

### Integrated analysis of chromatin accessibility and gene expression

To assess the relationship between the accessibility of individual peaks and the expression of nearby genes, peaks were assigned to genes by nearest TSS. In this way, differential expression of individual genes or the distribution of expression of groups of genes could be putatively associated with ATAC-seq peaks.

### GO enrichment analysis

Enrichment of GO Biological Process term within rod- or cone-specific ATAC seq peaks was performed using GREAT (v3.0.0)^86^.

### Motif enrichment analysis

Known motif enrichment and *de novo* motif discovery were performed for photoreceptor ATAC-seq peaks as well as brain, liver, lung and B cell DNase-seq peaks using HOMER (v4.8)^46^. Target sequences consisted of 200 bp elements centered on peak summits. Background sequences consisted of approximately 50,000 randomly selected 200 bp intervals from the mouse genome normalized for mono- and di-nucleotide content relative to each target set. Repeat sequences were masked from the genome, and targets with >70% of bases masked were dropped from enrichment analysis. Motif enrichment was performed separately for promoter peaks (peaks less than 1000 bp upstream and 100 bp downstream of an annotated TSS) and enhancer peaks. Sequence logos presented in Fig. 5 and Supplementary Fig. S7 were trimmed to remove flanking positions with low information content (<1 bit). Complete known and *de novo* motif enrichment results are presented in Supplementary Tables S7,S8,S9,S10. To assess motif co-occurrence, we first collapsed the database of 319 known motifs curated by HOMER to a set of 66 non-redundant motifs (by aligning motifs, calculating the PCC across nucleotide frequencies, and selecting the highest scoring motif among pairs with a PCC > 0.6). For each motif, we then counted the number of peaks with ≥1 occurrence, and for each pair, we counted the number of peaks with ≥1 pair. The enrichment of co-occurrence was then calculated as the log_2_(observed pairs/expected pairs), where the number of expected pairs was estimated from the frequency of individual motifs: expected = (number of peaks with motif 1) × (number of peaks with motif 2)/(total peaks). The pairwise co-occurrence enrichment matrix was plotted as a heatmap, with rows and columns clustered by Euclidean distance and average linkage. Preferential spacing between highly enriched motifs (K50 HD, MAF, bZIP, MADS, and NR motifs) was assessed by first centering shared photoreceptor ATAC-seq peaks on individual motifs, and then plotting the density of secondary motifs (using a relaxed log odds threshold of 5) on either strand upstream and downstream of the primary motif.

### Analysis of CRE-seq data

Previously generated CRE-seq data were downloaded from the supporting information included in White *et al*.^52^. Constructs were classified as having or not having k-mers of interest based on exact matches to TAAT (or ATTA), TAAG (or CTTA), TAAT…ATTA, TAAG…CTTA, or TAAT…CTTA (or TAAG…ATTA). We then examined the distribution of CRE-seq expression (a measure of the enhancer activity) based on the presence or absence of individual k-mers. In addition, CRE-seq constructs were determined to contain instances of motifs enriched in photoreceptor ATAC-seq peaks by scanning sequences for individual motifs (CRX, NEUROD1, MEF2D, and RORB) using HOMER (v4.8)^46^. We again examined the distribution of CRE-seq expression based on the presence or absence of individual motifs.

### Statistics and data visualization

Statistical analyses were implemented in R (v3.3.0)^87^ using base packages, as well as coin^88^ and DEseq2^56^. Data visualization was implemented with ggplot2^89^ and gplots^90^.

## Additional Information

**Accession Codes**: ATAC-seq data (raw data, signal tracks, peak calls, and count tables) and RNA-seq data (raw data, signal tracks, and count tables) have been deposited in the NCBI Gene Expression Omnibus (GEO) under accession number GSE83312.

**How to cite this article**: Hughes, A. E. O. *et al*. Cell Type-Specific Epigenomic Analysis Reveals a Uniquely Closed Chromatin Architecture in Mouse Rod Photoreceptors. *Sci. Rep.* **7**, 43184; doi: 10.1038/srep43184 (2017).

**Publisher's note:** Springer Nature remains neutral with regard to jurisdictional claims in published maps and institutional affiliations.

## Supplementary Material

Supplementary Information

Supplementary Table S1

Supplementary Table S2

Supplementary Table S3

Supplementary Table S4

Supplementary Table S5

Supplementary Table S6

Supplementary Table S7

Supplementary Table S8

Supplementary Table S9

Supplementary Table S10
