# Supplementary material for: Cell Type-Specific Epigenomic Analysis Reveals a Uniquely Closed Chromatin Architecture in Mouse Rod Photoreceptors
Source: Sci Rep. 2017 Mar 3;7:43184. doi: 10.1038/srep43184 (PMC5335693; doi:10.1038/srep43184)

SUPPLEMENTARY INFORMATION

SUPPLEMENTARY FIGURE LEGENDS

Supplementary Figure S1. Photoreceptor ATAC-seq and RNA-seq reproducibility. (*A-D*) Reproducibility between pairs of biological replicates for rod, *Nrl*$^{-/-}$ photoreceptor (blue cone), single-sorted green cone, and double-sorted green cone ATAC-seq. For each peak, the read count for biological replicate 1 (x-axis) is plotted against the read count for biological replicate 2 (y-axis). (*E-J*) Reproducibility between pairs of biological replicates for rod and *Nrl*$^{-/-}$ photoreceptor (blue cone) RNA-seq. For each gene, the read count for biological replicate 1 (x-axis) is plotted against the read count for biological replicate 2 (y-axis) (three pairs of replicates per cell type). PCC: Pearson correlation coefficient.

Supplementary Figure S2. Locus complexity of rod- and cone-specific genes. The 50 most differentially expressed genes between rods and blue cones (as assessed by RNA-seq) are ordered on the x-axis by fold-change (rod expression/cone expression). For each gene (column), each point represents an ATAC-seq peak that mapped to that gene (based on nearest TSS). Peaks shared by rods and cones are colored gray, rod-specific peaks are red, cone-specific peaks are blue. For rods, the bottom point for each gene is positioned on the y-axis to correspond to the estimated log of the fold change (rods/blue cones) in the expression of that gene; additional points (if present) are stacked on top. For cones, this value corresponds to the top point, and additional points (if present) are included beneath. Genes that are more highly expressed in rods are typically flanked by rod-specific peaks, whereas genes that are more highly expressed in cones are flanked by cone-specific peaks. Both rod- and cone-enriched genes are frequently flanked by shared ATAC-seq peaks. This style of data presentation was adapted from Gonzalez *et al.* 2015.

Supplementary Figure S3. Pairwise correlations between photoreceptor ATAC-seq and control tissue DNase-seq data. Peaks from each cell or tissue type were merged into a common set of 173,219 regulatory elements. Reads in each feature were counted in each cell type, and samples were clustered across features using Spearman's ρ with average linkage. Control tissues cluster distinctly from photoreceptors. Whole retina falls between brain and photoreceptors. Rods cluster distinctly from cones, and cone subtypes cluster together.

Supplementary Figure S4. Gene expression near shared (rod and cone), rod-specific, and cone-specific enhancer peaks. (*A*) Each photoreceptor enhancer ATAC-seq peak (n=35,173), was mapped to a gene based on nearest TSS. The distribution of expression for genes surrounding shared peaks was not significantly different in rods vs. cones. Gene expression was significantly higher in rods near rod-specific peaks (permutation test, 10,000 permutations). Gene expression was significantly higher in cones near cone-specific peaks (permutation test, 10,000 permutations). I.e., rod- and cone-specific ATAC-seq peaks are associated with elevated expression in rods and cones, respectively, but genes near cone-specific ATAC-seq peaks have lower expression in both cell types relative to genes near shared or rod-specific peaks. (*B*) Shared and rod-specific peaks were located approximately the same distance from genes, whereas cone-specific open chromatin was located significantly farther from genes (permutation test 10,000 permutations). In other words, regions selectively closed in rods are located significantly farther from genes than typical open chromatin elements.

Supplementary Figure S5. **Gene ontology (GO) enrichment for rod- and cone-specific ATAC-seq peaks.** (*A*) Rod-specific peaks were highly enriched for terms specifically related to vision and photoreceptor biology. (*B*) Cone-specific peaks were highly enriched for terms related to neurodevelopment and smooth muscle biology.

Supplementary Figure S6. Distinct features of photoreceptor promoters and enhancers. ATAC-seq peaks within -1 kb to +100 bp of a TSS were classified as "promoter" peaks, whereas peaks outside this

interval were classified as "enhancer" elements. (*A-B*) The average ATAC-seq signal (normalized reads per base pair) was higher in promoters compared to enhancers, and the enrichment over baseline was broader. (*C-D*) Mean conservation (phastCons 60-way vertebrate conservation) as well as GC content (E-F) were also higher and broader surrounding promoter peaks vs. enhancer peaks. (*G-H*) Promoter and enhancer peaks were mapped to genes by nearest TSS. In both rods and cones, the presence or absence of a promoter peak was more strongly correlated with gene expression than the presence or absence of an enhancer peak, but the presence of either was associated with increased expression.

Supplementary Figure S7. Enrichment of known TFBS motifs in promoter (TSS-proximal) peaks. For each panel, distance from peak summit (-500 bp to 500 bp) is plotted on the x-axis and motif density (motifs per peak at each position) is plotted on the y-axis, illustrating central enrichments of the motifs presented. Motifs are labeled by TF class, and the specific factor from which the sequence logo shown was derived is indicated in parenthesis. Compared to enhancers (Fig. 4A), motif enrichment in promoters is highly similar across cell and tissue types.

Supplementary Figure S8. **Motif co-occurrence in photoreceptor ATAC-seq peaks.** (*A*) Enrichment of motif co-occurrence was calculated for pairs of 60 known motifs based on the observed counts of pairs in peaks compared to the expected counts based on the frequency of individual motifs in peaks (Methods). Rows and columns are hierarchically clustered by Euclidean distance using average linkage. (*B*) The motifs from the boxed region in (*A*). bZIP, NR, MAF, MADS, and K50 HD TFs cluster together and are mutually co-enriched. These motifs are depleted of CTCF motifs (ZF, blue row and column).

Supplementary Figure S9. Preferential motif spacing flanking K50 HD sites. Each panel shows the strand-specific per nucleotide density of the indicated secondary motif upstream and downstream of K50 HD motifs in photoreceptor ATAC-seq peaks. Secondary motifs on the positive strand are shown in blue, above the midline. Secondary motifs on the negative strand are shown in red, below the midline.

SUPPLEMENTARY TABLES

Supplementary Table S1. Technical covariates and sequencing metrics for photoreceptor ATAC-seq and RNA-seq. RIN: RNA integrity number. Raw sequencing reads: number of forward and reverse reads for each sample. Processed sequencing reads: number of forward and reverse reads after filtering out improperly paired reads, reads with mapping quality <30, reads aligning to the mitochondrial genome, reads aligning to unplaced or unlocalized contigs, ENCODE blacklist regions, and PCR duplicates.

Supplementary Table S2. Annotated ATAC-seq peaks. Consensus peak calls for each cell type are presented on separate worksheets.

Supplementary Table S3. Overlap between photoreceptor ATAC-seq and additional whole-retina epigenomic datasets.

Supplementary Table S4. Datasets and accessions.

Supplementary Table S5. Differentially accessible peaks (rods vs. cones). Rod 1, rod 2, green cone 1, green cone 2, blue cone 1, blue cone 2: normalized ATAC-seq reads for each cell type. Base mean: average across all cell types. Log_2(fold change): fold change is calculated as rod ATAC-seq reads / cone ATAC-seq reads. Green cones and blue cones are collapsed into a single level for statistical analysis.

Supplementary Table S6. Differentially expressed genes (rods vs. blue cones). Rod 1, rod 2, rod 3, blue cone 1, blue cone 2, blue cone 3: normalized RNA-seq reads for each cell type. Base mean: average across all cell types. Log_2(fold change): fold change is calculated as rod RNA-seq reads / blue cone RNA-seq

reads.

Supplementary Table S7. Known motif enrichment: photoreceptor promoters. A complete list of sequence logos and position weight matrices (PWMs) for individual motifs is available online in the HOMER motif database: http://homer.salk.edu/homer/motif/HomerMotifDB/homerResults.html.

Supplementary Table S8. Known motif enrichment: photoreceptor enhancers. A complete list of sequence logos and position weight matrices (PWMs) for individual motifs is available online in the HOMER motif database: http://homer.salk.edu/homer/motif/HomerMotifDB/homerResults.html.

Supplementary Table S9. *De novo* motif enrichment: photoreceptor promoters. PWMs for *de novo motifs* are included as a separate worksheet.

Supplementary Table S10. *De novo* motif enrichment: photoreceptor enhancers. PWMs for *de novo motifs* are included as a separate worksheet.

Supplementary Fig. S1

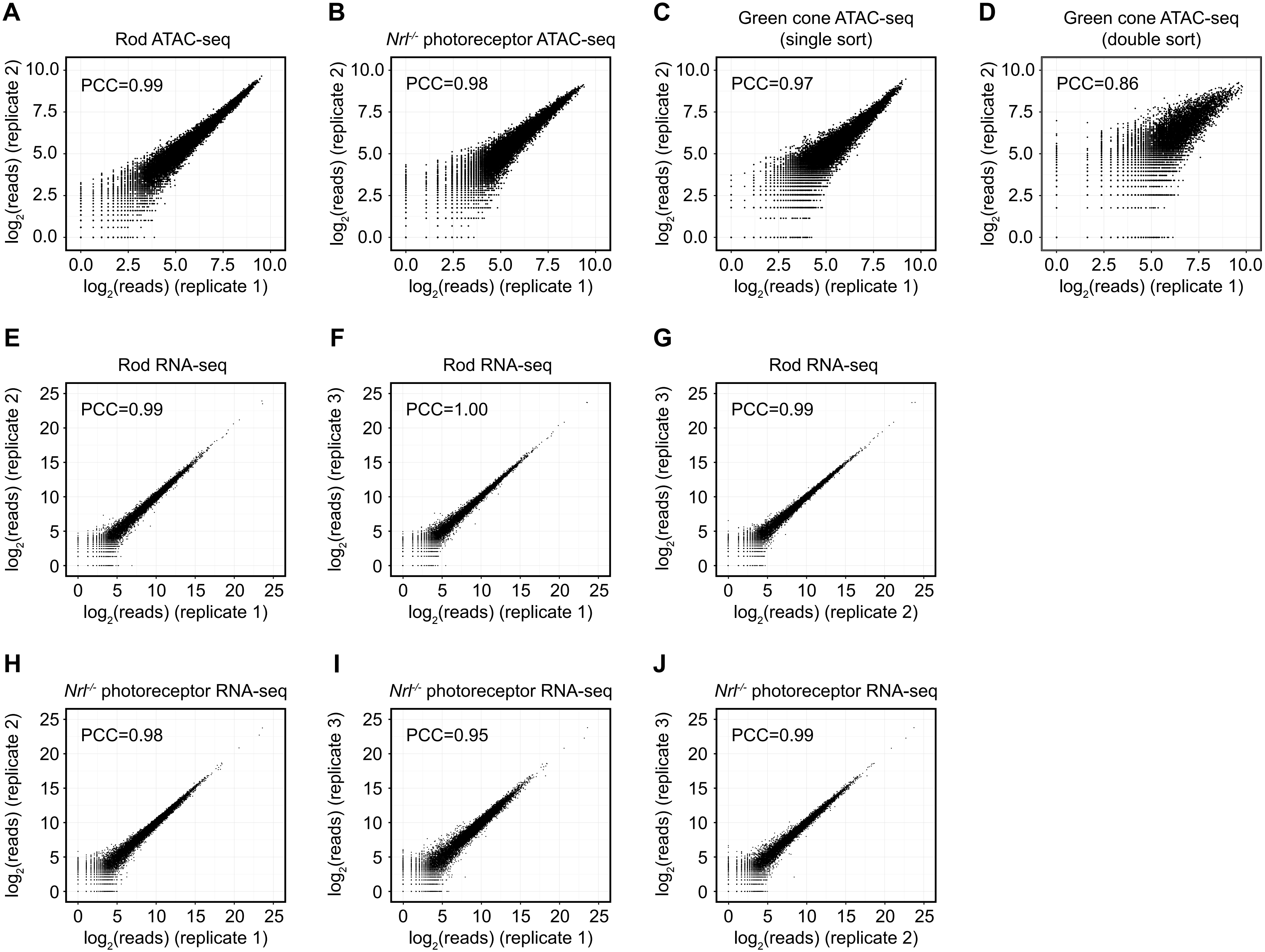

Supplementary Fig. S2

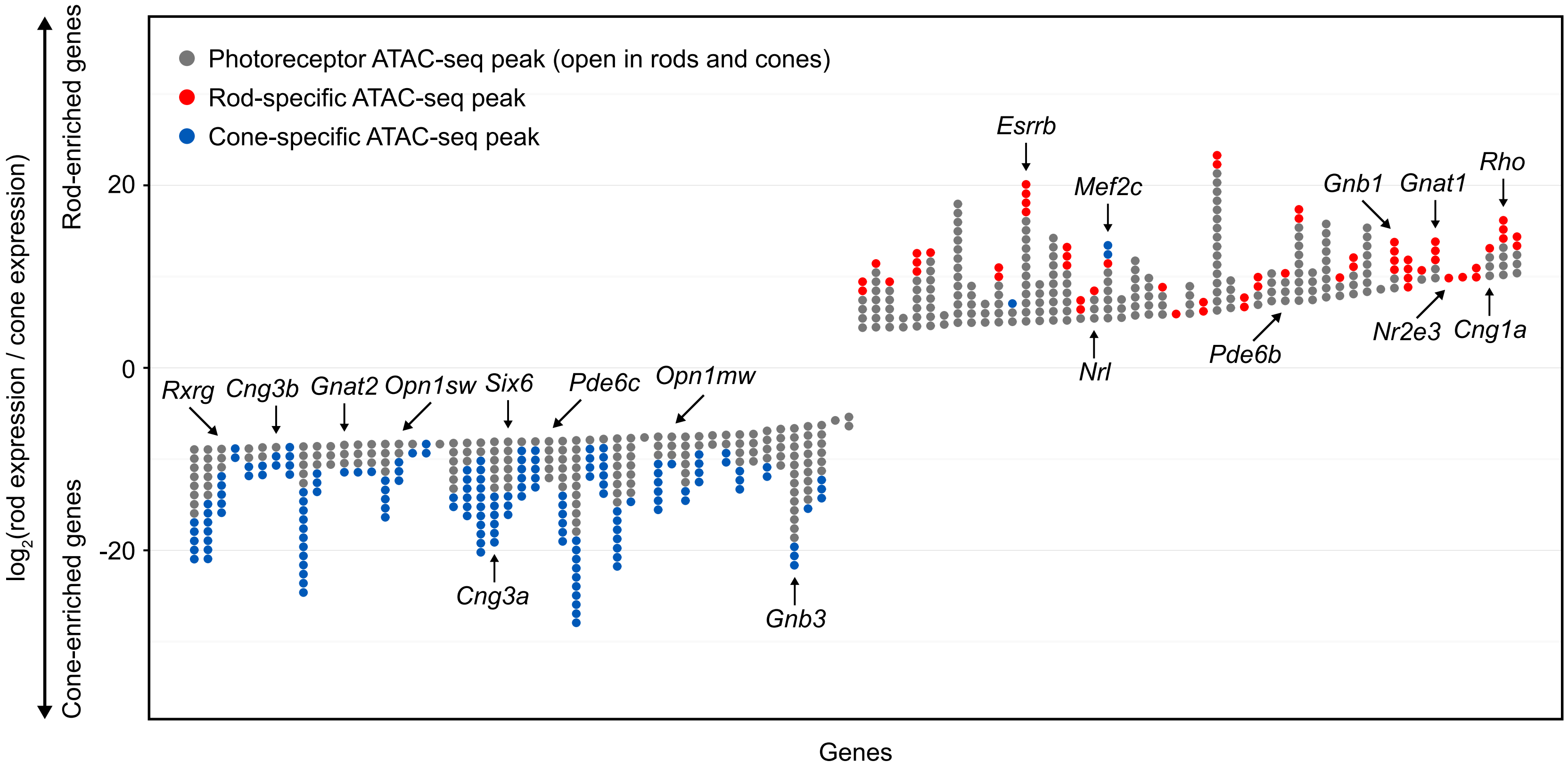

Supplementary Fig. S3

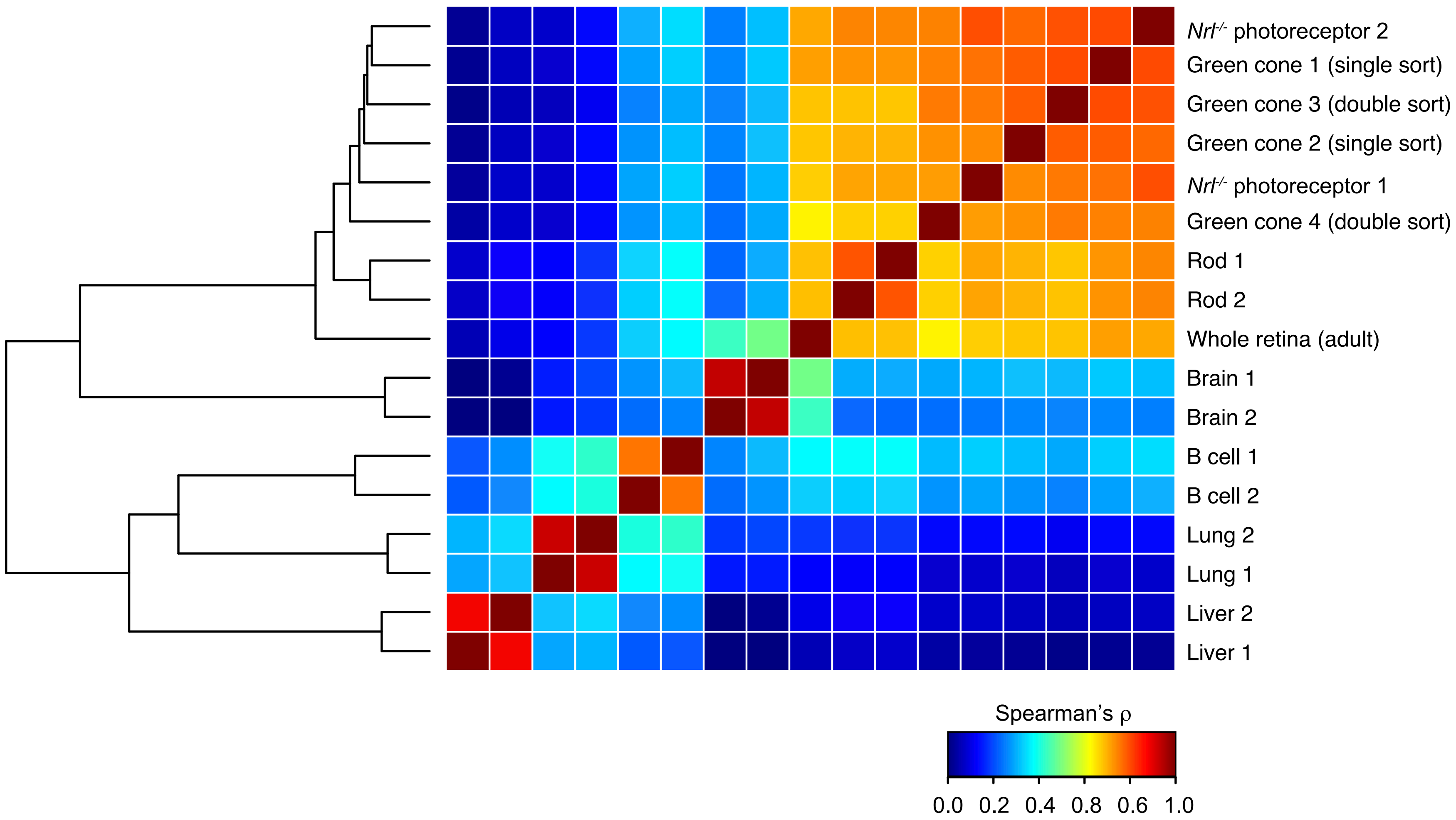

Supplementary Fig. S4

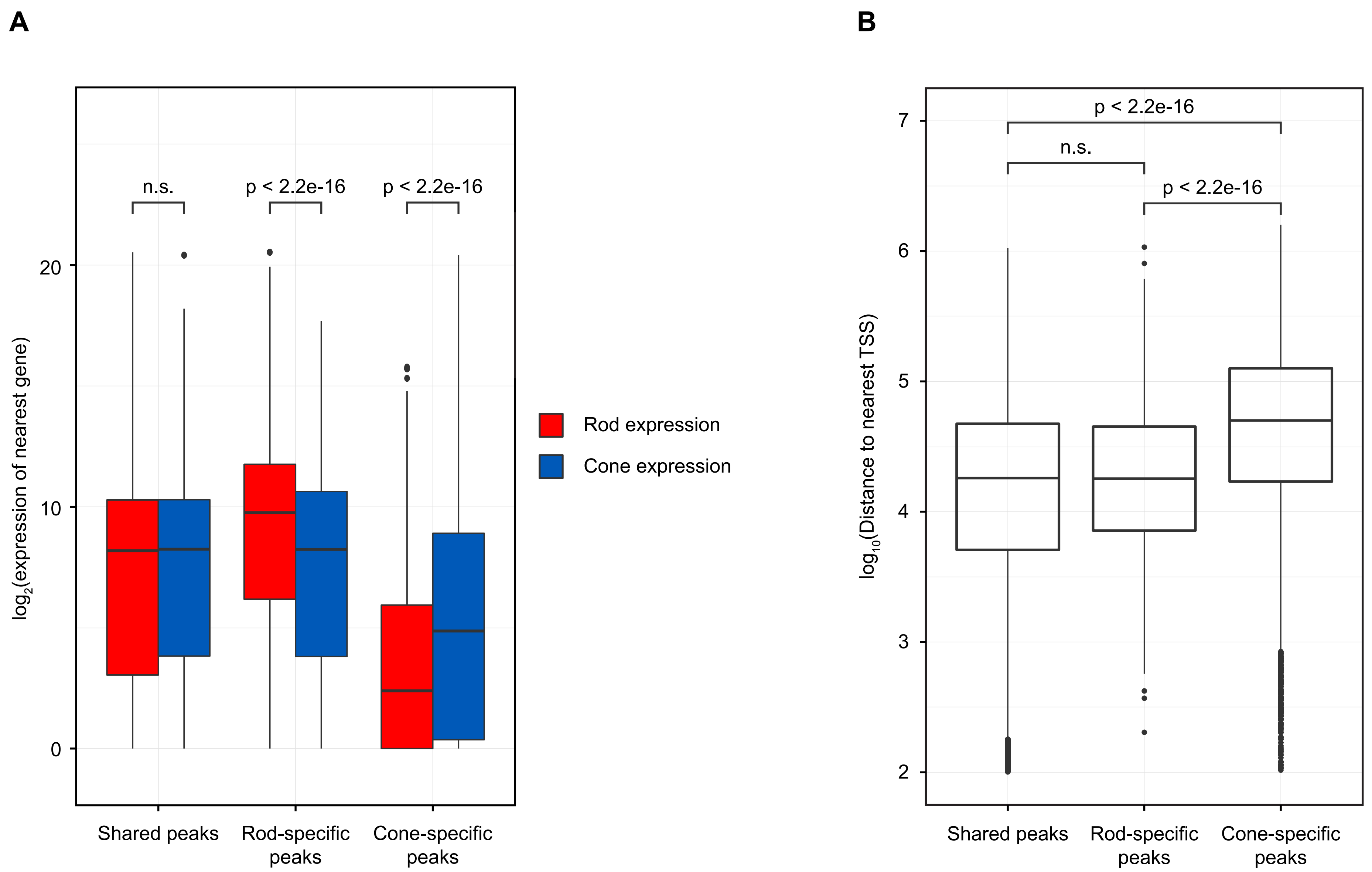

Supplementary Fig. S5

A

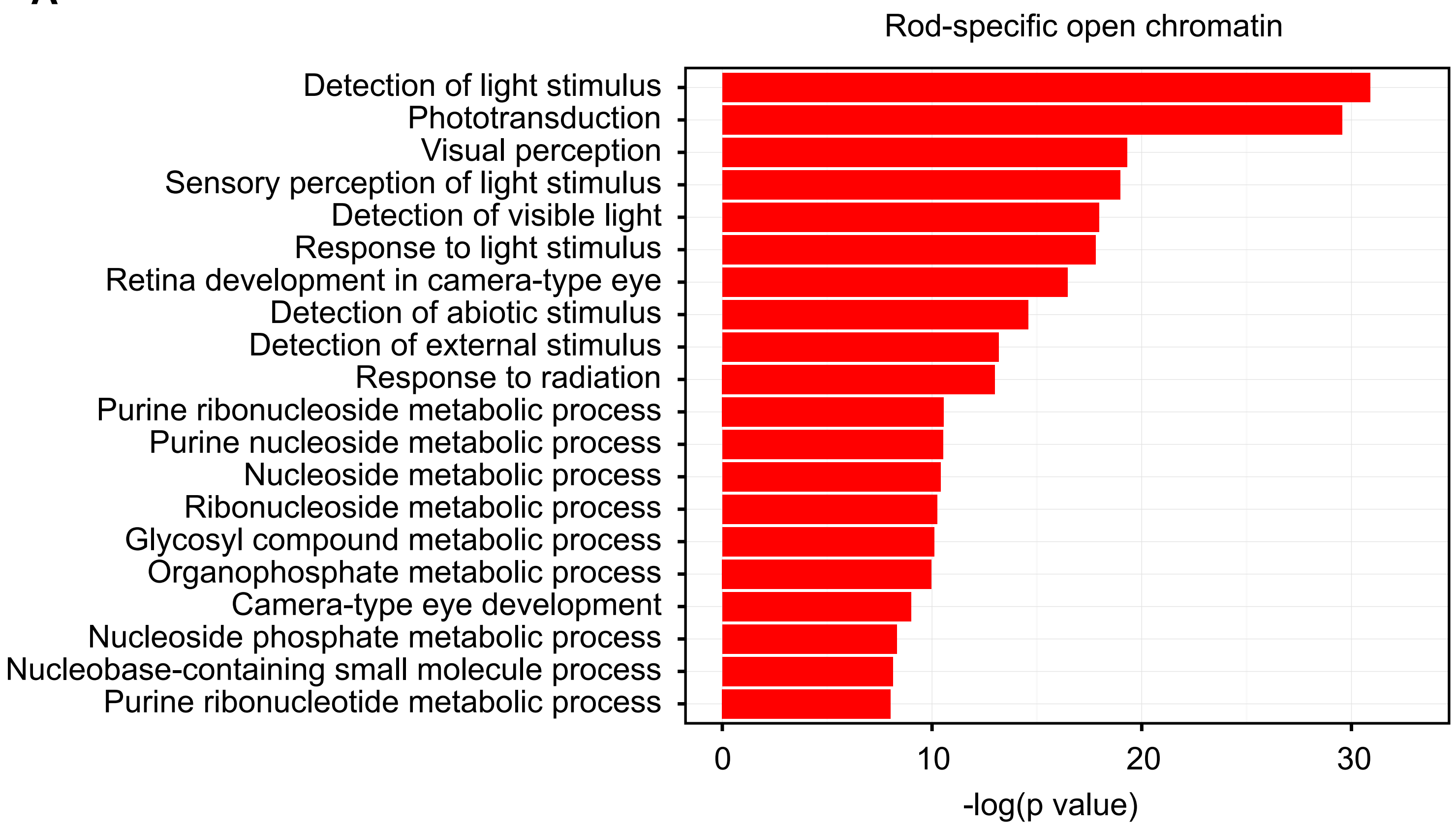

B

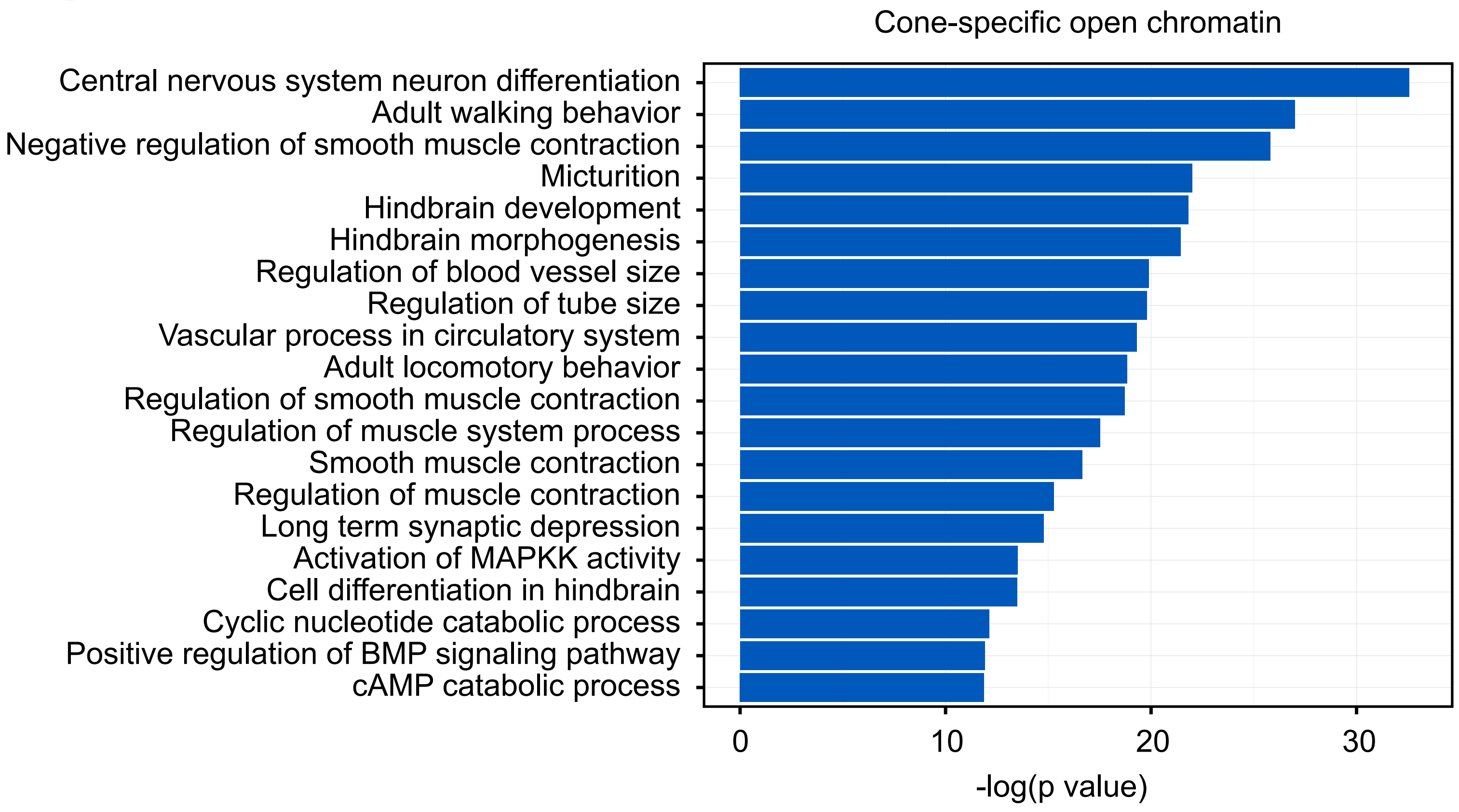

Supplementary Fig. S6

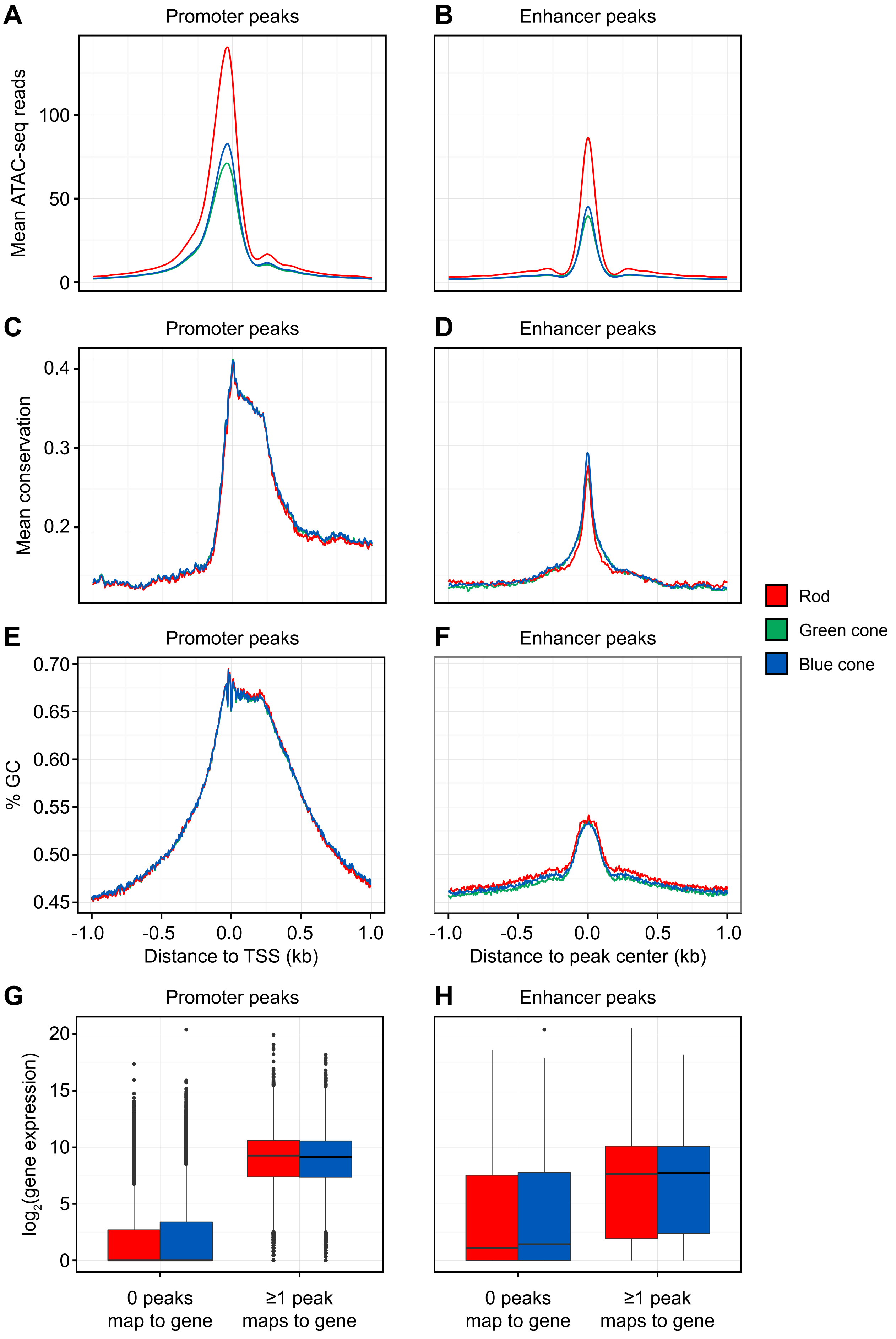

Supplementary Fig. S7

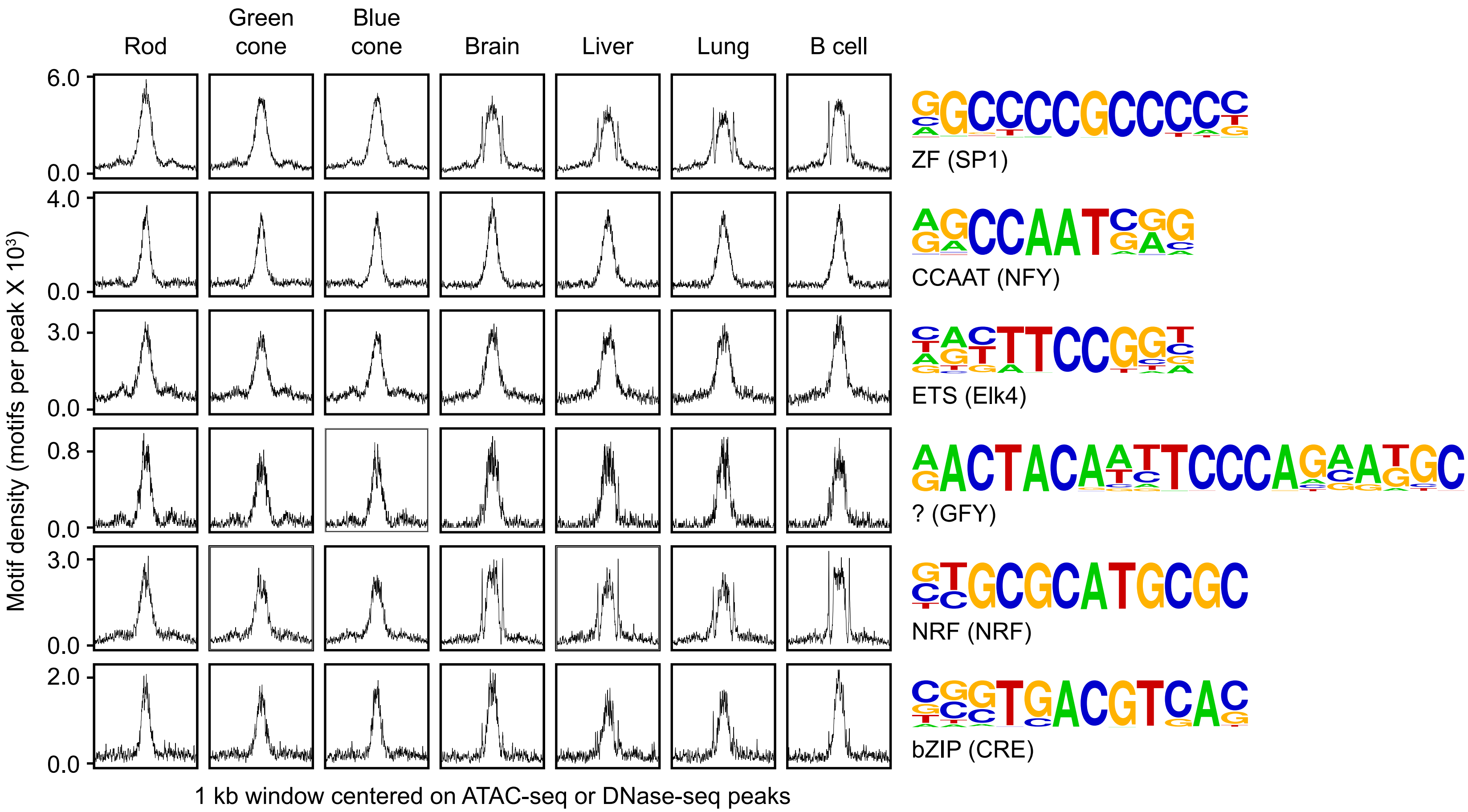

Supplementary Fig. S8

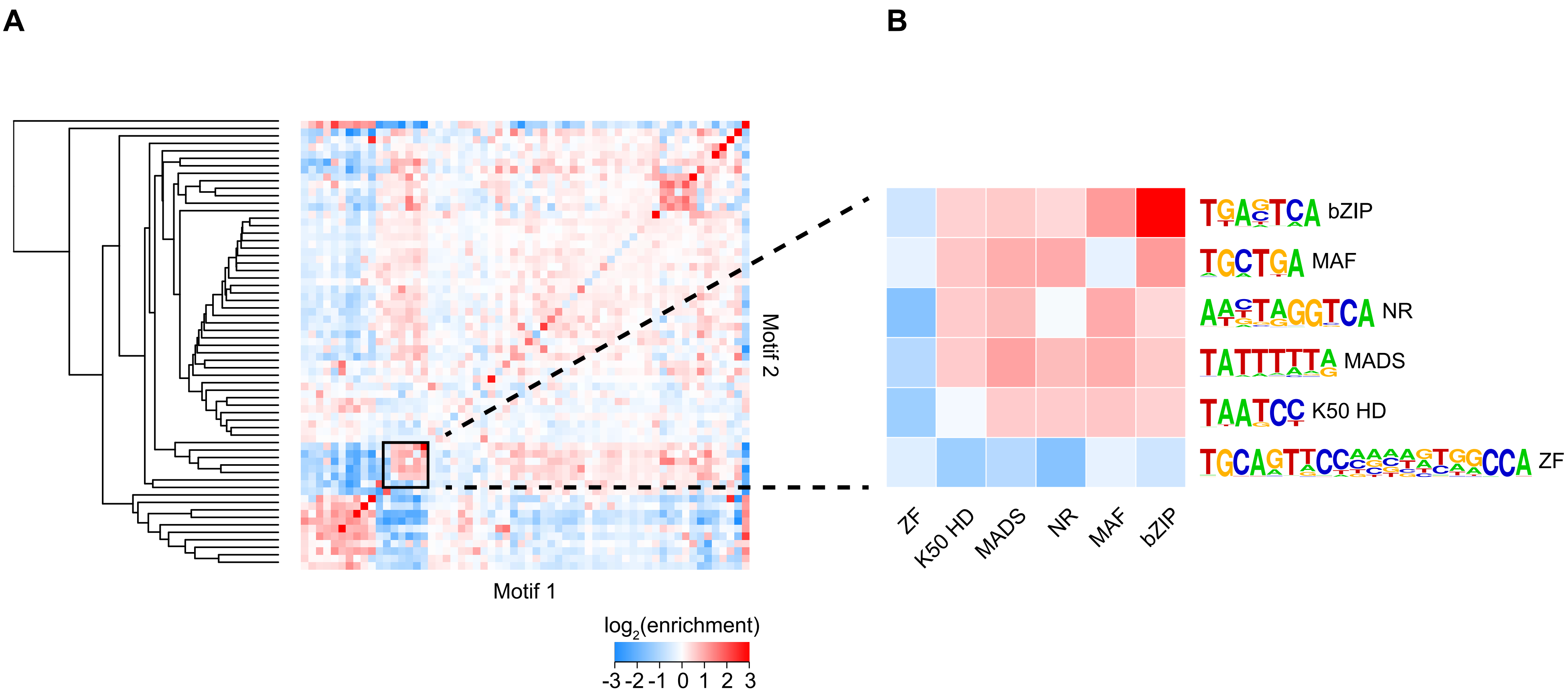

Supplementary Fig. S9

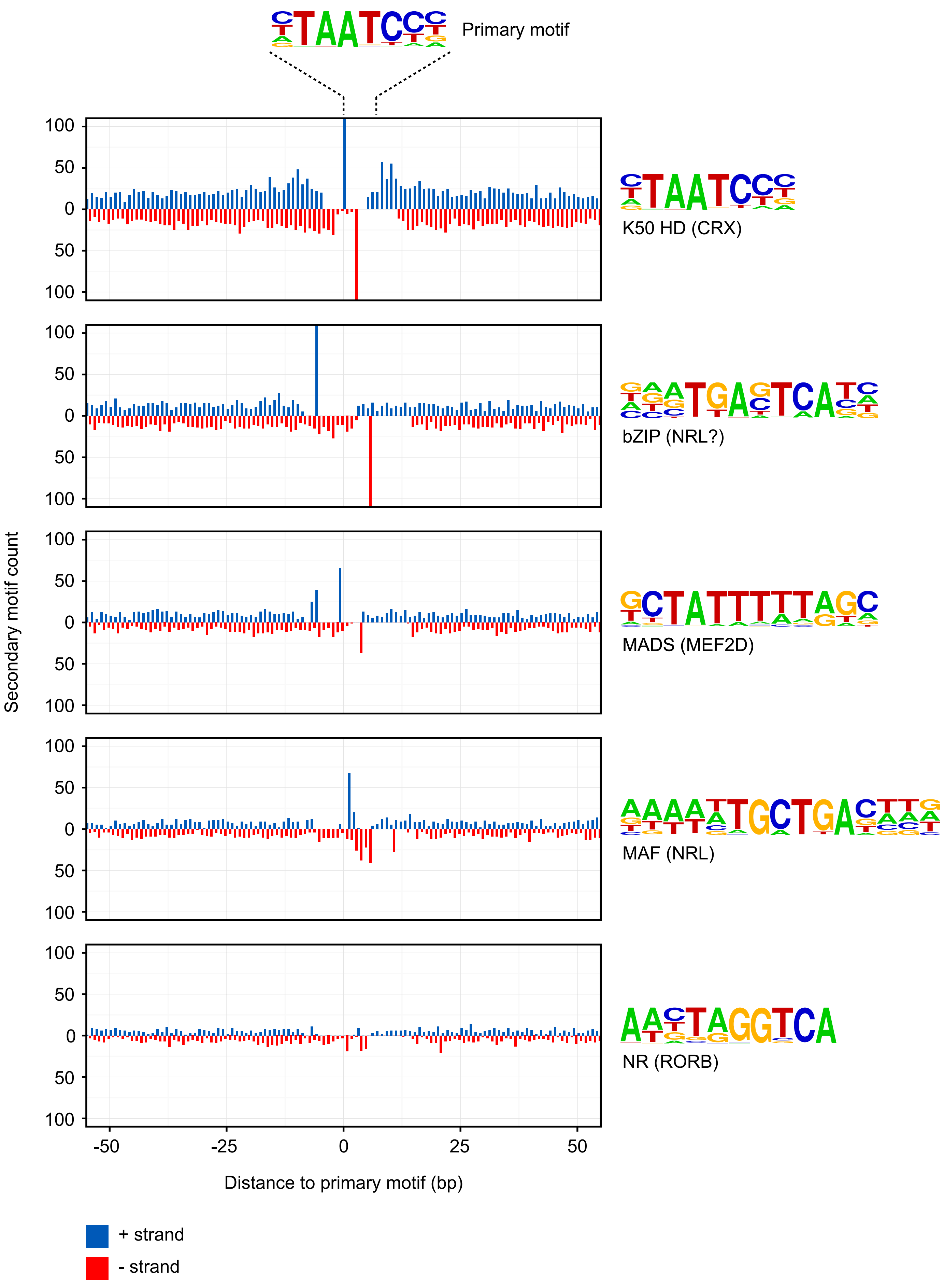

Supplement: Supplementary Information [file srep43184-s1.pdf]
